# Synthesis, Anti-Influenza H1N1 and Anti-Dengue Activity of A-Ring Modified Oleanonic Acid Polyamine Derivatives

**DOI:** 10.3390/molecules27238499

**Published:** 2022-12-02

**Authors:** Irina Smirnova, Anastasiya Petrova, Gul’nara Giniyatullina, Anna Smirnova, Alexandrina Volobueva, Julia Pavlyukova, Vladimir Zarubaev, Tran Van Loc, Thao Tran Thi Phoung, Vu Thi Bich Hau, Nguyen Thi Thu Thuy, Myint Myint Khine, Oxana Kazakova

**Affiliations:** 1 Ufa Institute of Chemistry, The Ufa Federal Research Centre, The Russian Academy of Sciences, 71 Prospect Oktyabrya, Ufa 450054, Russia; 2 Department of Virology, St. Petersburg Pasteur Institute of Epidemiology and Microbiology, Experimental Virology Laboratory, 14 Mira St., St. Petersburg 197001, Russia; 3 St. Petersburg State Institute of Technology, 26 Moskovsky Av, St. Petersburg 190013, Russia; 4 Institute of Chemistry, Vietnamese Academy of Science and Technology, 18-Hoang Quoc Viet Street, Cau Giay District, Hanoi 1000, Vietnam; 5 National Institute of Hygiene and Epidemiology, 1 Yersin Street, Hai Ba Trung, Hanoi 10000, Vietnam; 6 Department of Chemistry, University of Yangon, University Avenue Road, Kamayut Township, Yangon 11041, Myanmar

**Keywords:** triterpenoids, oleanolic acid, polyamine, Ugi derivatives, indole, benzylidene, antiviral activity, influenza A (H1N1), Dengue virus

## Abstract

A series of sixteen A-ring modified (2,3-indolo-, 2-benzylidene) oleanonic acid derivatives, holding some cyclic amines, linear polyamines and benzylaminocarboxamides at C28, has been synthesized and screened for antiviral activity against influenza A/PuertoRico/8/34 (H1N1) and Dengue virus serotypes of DENV-1, -2, -3, -4. It was found that 28-homopiperazine **2** and 3-*N*-phthalyl **22** amides of oleanonic acid demonstrated high potency with selectivity index SI 27 (IC_50_ 21 μM) and 42 (IC_50_ 12 μM). Oleanonic acid aminoethylpiperazine amide **6** and C-azepano-erythrodiol **23** appeared to be the most effective compounds against DENV-1 (IC_50′_s 67 and 107 μM) and -2 (IC_50′_s 86 and 68 μM correspondingly) serotypes.

## 1. Introduction

To date, influenza, HIV, SARS, MERS, COVID-19, and Dengue have become the leading viral diseases threatening human health. Triterpenes and its semisynthetic derivatives are compounds with a promising antiviral potential because of their significant pharmaceutical values, broad spectrum of applications towards antiviral medication, low cost, high efficacy and low side effects [1,2,3,4]. Great progress in their discovery, isolation, and especially in the elucidation of their complex structures, was made at the beginning and in the middle of the 20th century, with the first partial syntheses taking place in this period [5]. Oleanolic acid (OA) is a pentacyclic triterpenic acid that is widely distributed in various plant foods and medicinal plants of Chinese traditional herbal medicines, including those growing in the regions of the East and Southeast Asia, including China, such as *Achyranthes aspera* [6], *Monotheca buxifolia* [7], *Ocimum sanctum* [8]. OA possesses a wide spectra of pharmacological activities, such as antimicrobial activities against a broad range of pathogens, anti-oxidant, antifungal, antidiabetic and have been shown to be clinically effective anti-inflammatory agents [9]. It has also been used as a drug for the liver in China, due to its hepatoprotective effect [10]. It is also effective against various spectrum of tumor cells and causes cell cycle arrest and apoptosis in human hepatocellular carcinoma cells [11]. In addition, OA and its derivatives possess notable antiviral activity against HIV [12]. OA extracted from *Leucas cephalotes* showed activity against the Dengue virus of the 2 serotype in the in vitro assays [13].

Among the numerous OA chemical derivatives, conjugates with amino acids, diamines and polyamines resulted in a series of antiviral, anticancer and antimicrobial agents [14]. Recently, 3-*O*-β-chacotriosyl oleanolic acid benzyl ester has been identified as a novel low molecular weight inhibitor of the SARS-CoV-2 virus [15]; while 3β- and 3α-amino-derivatives of oleanolic acid were shown to possess inhibitory activity against HCV protease [16]. OA-arginine conjugate exhibited robust potency and broad antiviral spectrum with IC_50_ values in low-micromolar range against four different influenza strains, including the oseltamivir-resistant strain A/Texas/50/2012 and influenza B viruses (BX-35 and BX-51B) [17]. In addition, OA-glucose conjugate displayed the highest anti-influenza A/WSN/33 activity with an IC_50_ of 5.47 μM and demonstrated no obvious cytotoxic effect on MDCK cells at 100 μM. [18]. Some OA trimers, prepared via the copper-catalyzed azide–alkyne cycloaddition reaction, were observed to exhibit robust potency against influenza A/WSN/33 (H1N1) [19]. Molecular ribbons consisting of a multifunctional PEG3 unit with a 1,2,3-triazole ring and an amide bond between oleanolic acid molecules showed cytotoxicity in the malignant melanoma cancer cell line [20]. OA amides with picolylamines, 2-dimethylaminoethylamine and 1-(2-aminoethyl)-pyrrolidine displayed fair-to-high cytotoxicity for several human tumor cell lines, with low EC_50′_s [21]. Amine- and guanidine-functionalized derivatives of betulinic, ursolic, and oleanolic acids showed considerably high bacteriostatic activity against methicillin-resistant *S. aureus* and exhibited excellent antifungal activity against *C. neoformans* [22]. Conjugates of oleanolic, asiatic and betulinic acids with spermine at the C3 and C28 positions through a succinate spacer showed not only antimicrobial and antitumor activity [23,24], but also self-assembled into J-type fibrous systems in the aqueous media, and also form supramolecular networks, thus opening up many possibilities for the use of such structures for drug delivery systems in serum or other body fluids [24,25]. Oleanonic acid conjugate with diethylentriamine was active against *S. aureus* (MIC 12.5 μM) and against *E. coli* and *P. aeruginosa* (MICs 25 and 50 μM, respectively) [26]. The long chain polyamine derivatives of oleanolic acid at C3 presented high to moderate activity against Gram-positive *S. aureus*, *S. faecalis* and *B. cereus* bacteria, with minimal inhibitory concentration (MIC) values from 3.125 to 200 µg/mL, and possessed important antimicrobial activities against Gram-negative *E. coli*, *P. aeruginosa*, *S. enterica*, and EA289 bacteria with MICs ranging between 6.25 and 200 µg/mL. The lead *N*-methyl-nor-spermidine conjugate OA showed the effect of disruption of the outer bacterial membrane of *P. aeruginosa* PA01 cells [27].

Coupling polyamines, in particular spermine, with an oleanolic acid frame, lead to improved biological potency, water solubility and bioavailability [28,29]. Taking into account these data, we have concluded that triterpenic acids used in conjugation with polyamines usually have a native scaffold or C3-acetyl-substituent. The antiviral potency of A-ring modified oleanolic acid conjugates with polyamines has seldom, if ever, been studied. It is known that the introduction of indole or benzylidene fragments into the triterpene core improves anticancer activity [30,31,32]. The cytotoxic properties of 2,3-indolo-oleanonic acid [33] and C2-[4-pyridinylidene]-oleanonic amides [34] have been described.

Therefore, taking into account the high potency of OA as an antibacterial and antiviral scaffold [26,34,35], we present a synthesis of new oleanonic, 2,3-indolo-and 2-pyridinoylidene-oleanonic acids dipeptides and conjugates with some linear and cyclic polyamines, as well as a screening of their antiviral activity against influenza A (H1N1) and all serotypes of Dengue virus.

## 2. Results and Discussion

### 2.1. Chemistry

**Scheme 1:**
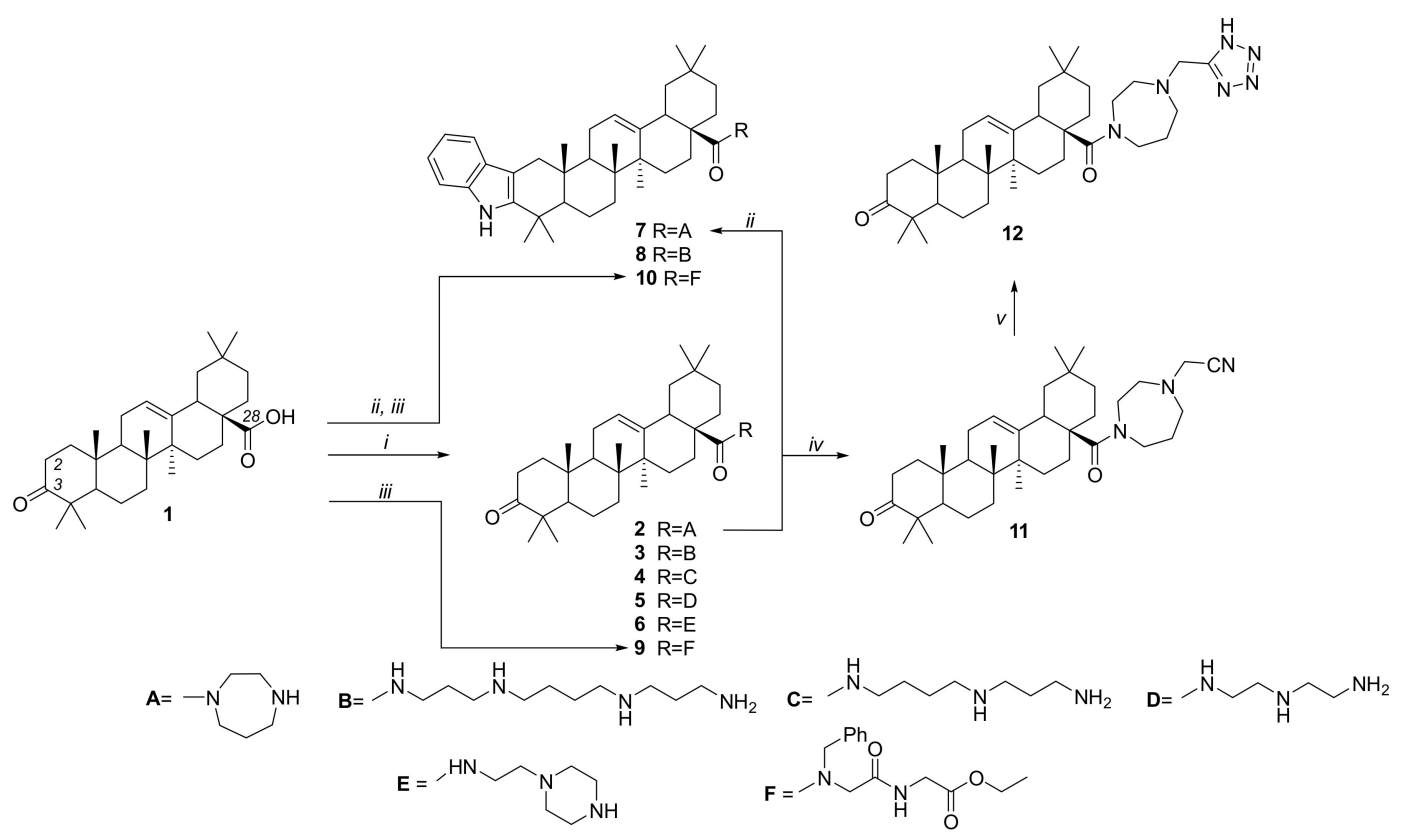
Reagent and conditions: i. a. (COCl)_2_, Et_3_N, CH_2_Cl_2_, 25 °C; b. gomopiperazine (for A), spermine (for B), spermidine (for C), diethylentriamine (for D), or aminoethylpiperazine (for E), Et_3_N, CH_2_Cl_2_, reflux; ii. PhNHNH_2_, AcOH, reflux; iii. BnNH_2_, paraform, CNCH_2_COOEt, 25 °C; PhNHNH_2_, AcOH, reflux; iv. ClCH_2_CN, K_2_CO_3_, DMF, reflux; v. NaN_3_, NH_4_Cl, DMF, reflux.

Oleanonic acid **1** was used as a starting platform for chemical modification. A series of diverse oleanonic acid amides **2**–**4**, **6** were prepared from **1** by condensation with acid clorides using homopiperazine, spermine, spermidine, diethylentriamine or aminoethylpiperazine to afford the corresponding amides **2**–**4**, **6** in 51–83% yield after purification by column chromatography (Scheme 1). The compound **5** with aminoethylpiperazine fragment was obtained earlier, according to the method [26]. The formation of compounds was established by NMR measurement. According to the ^13^C NMR spectra, the peaks of the C28 were located at δ 175.6–178.3 ppm (compared δ 181.0 ppm for **1**), showing the formation of amide bond. In the ^1^H NMR spectra, the new signals of methylene protons of the alkane-polyamine moieties as multiplets were located at δ 2.47–3.53 ppm of the homopiperazine fragment, at δ 2.26–3.28 ppm, correspondently. The signals of the protons of the aminomethylene groups of compound **6** appeared at δ 2.83–3.46 ppm (Supplementary Material, Figures S1–S8). By the Fischer indole formation of amides **2** and **3** with phenylhydrazine, indoles **7** and **8** were obtained in 59–65 % yields. The signals of indole moiety appeared in the ^1^H NMR spectrum at δ 6.97–7.54 ppm and in the ^13^C NMR spectrum at δ 110.3–136.2 ppm; the signals of C2 and C3 appeared at δ 106.9 and 140.9 (140.8) ppm, respectively. In the initial compounds **2** and **3**, the characteristic signal of the carbonyl carbon in the C3 position is located at δ 217.6 (217.8) ppm (^13^C NMR). Compounds **9** and **10** were obtained through a Ugi multicomponent reaction of oleanonic acid **1** or 2,3-indolo-oleanolic acid with benzylamine, paraform and ethyl 2-isocyanoacetate with 75% and 71% yields, respectively. Compared to the spectra of parent compounds, which contained only one signal of the carboxyl group at δ 180–181 ppm, the ^13^C NMR spectra of compounds **9** and **10** exhibited three signals of carbonyl groups, at δ 169 (C(O)O), 170 (C(O)NH) and 178 (C(O)N) ppm (Supplementary Material, Figures S9–S16). Oleanonic homopiperazine amide **2** was functionalized at a free *N*H-group by the alkylation with chloroacetonitrile using a procedure [36] affording cyano-derivative **11**, which was confirmed by a signal of CN-group at δ 115.2 ppm (^13^C NMR) and methylene group at δ 4.39–4.61 ppm (^1^H NMR). Moreover, tetrazole **12** was successfully synthesized by refluxing compound **11** with NaN_3_ in the presence of ammonium chloride. In the ^13^C NMR spectrum, the signal of the C9′ atom was observed at δ 162.7 ppm (^13^C NMR) (Supplementary Material, Figures S17–S20).

**Scheme 2:**
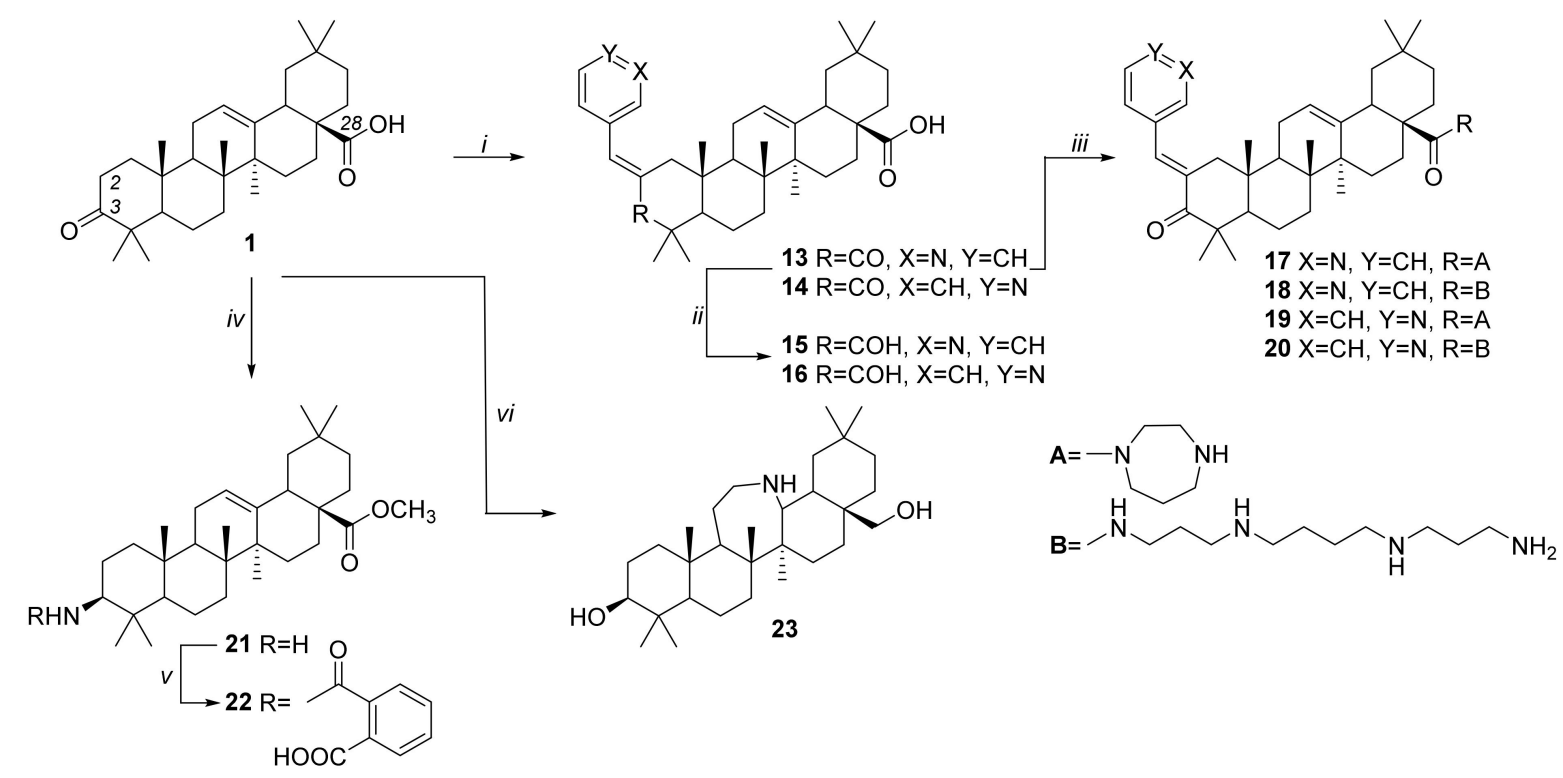
Reagent and conditions: i aldehydes, 40%KOH/EtOH, EtOH, 22 °C, ii. NaBH_4_, MeOH, 25 °C; iii. a. (COCl)_2_, Et_3_N, CH_2_Cl_2_, 25 °C; b. gomopiperazine (for A), spermine (for B), Et_3_N, CH_2_Cl_2_, reflux; iv. NH_4_OAc, NaBH_3_CN, MeOH, 22 °C; v. phthalic anhydride, DCC, DMAP, CH_2_Cl_2_, 0 °C; vi. a. NaBH_4_, MeOH, rt; b. AcCl, pyridine, reflux; c. O_3_, CH_2_Cl_2_, −40 °C; d. NH_2_OH∙HCl, pyridine, reflux 4 h; e. SOCl_2_, 1,4-dioxane, 22 °C, 30 min; f. LiAlH_4_, 1,4-dioxane, reflux, 14 h.

The synthesis of the A-ring modified oleanonic acid amides linked with the C28 position is presented in Scheme 2. The Claisen-Schmidt reaction of the oleanonic acid **1** with 3- or 4-pyridinecarboxaldehydes afforded C2-nicotinoylidene derivatives **13** and **14**. The 3*β*-Hydroxy-derivatives **15** and **16** were obtained by the reduction in the 3-oxo-group of **13** and **14** NaBH_4_ reduction in methanol, which is confirmed by the up-field shift of the signal of the C3 carbonyl atom from δ 199.9 (200.3) ppm (^13^C NMR) (for parent compounds **13**, **14** [37]) to δ 80.8 (80.9) ppm (^13^C NMR) (Supplementary Material, Figures S21–S24). The interaction of the C2-nicotinoylidene derivatives **13** or **14** with homopiperazine or spermidine led to amides **17**–**20** (Supplementary Material, Figures S25–S32) in 52–86% yields. Methyl 3*β*-amino-oleanoate **21** was acylated by phthalic anhydride using the DCC method to form compound **22** in high yield. In the spectrum, the download shift of the signal of carbon atom in the C3 position from δ 59.7 ppm (for compound **21** [38]) to 61.1 ppm (^13^C NMR) was observed; the signals of the CONH group, at δ 169.5 ppm (^13^C NMR), and the signals of the phthalic fragment at δ 134.0, were located at 123.0 ppm (^13^C NMR) and 7.61–7.90 ppm (^1^H NMR), respectively, while a free carboxylic group was located at δ 169.2 ppm (^13^C NMR) (Supplementary Material Figures S33, S34). The compound **23** was obtained according to [35].

### 2.2. Anti-Influenza A H7N1 Virus Activity

Based on the analysis of the Comprehensive Medicinal Chemistry Database, more than 25% of known drugs contain a carboxamide as a structural feature [39]. The literature data showed that nitrogen-containing triterpenoids with a modified A-ring led to an increase in antiviral activity, and in particular, anti-influenza activity [40]. These results primarily include amide functions at C28 positions. Thus, betulonic acid *L*-leucine-amide demonstrated 90% suppression of the influenza virus A H7N1 (EC_50_ 2.17, MTT/EC_50_ 392.16) [41,42]. Lupane 3,4-seco-3,28-bis-methylpiperazine-amide showed IC_50_ 27 µM and SI 7.1 against the Flu A H1N1 strain [43]. The Lupane-type conjugate with 2-aminopropane-1,3-diol was active against herpes simplex virus type I (5.7 μM, MTC/EC_50_ 32.2) [44].

Initially, maximum non-toxic concentrations were determined for compounds **1**–**22** to exclude their non-specific antiviral activity. The in vitro cytotoxicity of the tested compounds (CC_50_) was studied in the normal Madin-Darby Canine Kidney cell line (MDCK) by the MTT ((3-(4,5-dimethylthiazol-2-yl)-2,5-diphenyltetrazolium bromide) test. The highest concentration tested was 100 µg/mL. The cytotoxicity of oseltamivir carboxylate and rimantadine was also evaluated as these have been used as reference drugs in antiviral tests. Compounds at concentration that kills no more than 50% of cells (CC_50_) were used for further investigation of antiviral activity.

The cytotoxic activity of some compounds was lower than that of the reference drugs used. Thus, oleanonic acid homopiperazine amide **2**, aminoethylpiperazine amide **6**, Ugi derivative **11** and *N*-phthalyl-amide **22** did not show cytotoxicity with CC_50_ > 560–437 µM (Table 1). Interestingly, spermidine amide **8** (CC_50_ > 140 µM) displayed a 7-fold lower cytotoxicity than diethylenetriamine-amide **5** with CC_50_ 48 µM, thus demonstrating the influence of a length of the side chain. A similar observation was made by Gupta et al. in [45], showing that OA derivatives with a long-chain alkyl carboxamides possess low cytotoxicity among other triterpenic acids. Among the Ugi derivatives, the presence of an indole fragment in the A-ring (compound **10**), in contrast to amide **9**, leads to a 3-fold increase in toxicity.

Anti-influenza properties of the selected derivatives **2**, **6**, **8**–**11** and **22** were studied in a MDCK cell culture against influenza virus A/PuertoRico/8/34 (H1N1). The resulting data, expressed as virus inhibiting activity (IC_50_), as well as selectivity index (SI), which is the ratio CC_50_/IC_50_, are presented in Table 1. The tested compounds showed strong differences in their antiviral activity. The moderately active compounds gomopiperazine amide **2** and aminoethylpiperazine amide **5** showed similar good antiviral activity with IC_50_ values of 20–21 µM and were roughly at the level of the reference drug rimantadine (IC_50_ 61 µM). The introduction of a long-chain amide spermine fragment in the OA scaffold (compound **8**), as well as ethylpiperazine fragment (compound **6**), led to the decrease in antiviral activity (IC_50_ values 27 and 82 µM, respectively). Amide **6** with an aminoethylpiperazine substituent showed the lowest virus-inhibiting activity, with an IC_50_ value of 82 µM. For these two compounds, **6** and **8**, the selectivity index SI was 5. Compounds **9**, **10** and **11** showed a similar level of anti-influenza activity with an IC_50_ value of 41, 39 and 45 µM and SI’s > 11, 3 and >12, respectively. Thus, gomopiperazine amide **2** with IC_50_ of 21 µM and SI > 27 was established as the most active compound in this series, which is consistent with the data, demonstrating that triterpenoids derivatives with cyclic amide moieties had the highest antiviral activity [46]. Previously, we have found that methyl 3*β*-amino-oleanoate **21** has antiviral activity with an IC_50_ of 7.9 μM, but a higher CC_50_ of 18 μM which led to lowering the selectivity index (SI 2) [47]. The esterification of methyl 3*β*-amino-oleanoate **21** with phthalic anhydride resulted increased activity (IC_50_ of 12 μM), but significantly lower toxicity (CC_50_ > 486 μM) in compound **22**, resulting in a selectivity index of >42. In this series of OA derivatives, compound **22**, due to its low toxicity and highest inhibitory activity, is defined as the lead-compound which showed the strongest anti-influenza potential [48]. It has previously been shown that the presence of a phthalic substituent in the structure of lupane triterpenoids also plays a crucial role in the presence of antiviral activity against flu A H1N1, H7N1, H3N2 and B viruses [41].

Importantly, the virus used in our study was, similarly to the majority of currently circulating influenza viruses, resistant to adamantane derivatives due to S31N substitution in M2 protein. This results in the low activity of the reference compound Rimantadine (IC_50_ = 6 μM, SI = 6). The high activity of the triterpene acids studied here therefore suggests that their target differs from the M2 proton channel and allows us to consider them as potential antivirals with an alternative mechanism of activity.

### 2.3. Anti-Dengue Activity

The anti-Dengue activity of native triterpenic acids was established for ursolic [49], oleanolic [13], glycyrrhizic [50] acids, which reduced the viral infectivity of the DENV-2 type with the IC_50_ of 8.1 μM. With regard to the triterpenic derivatives, we have found very limited literature data on their suppressing activity against the Dengue virus. Thus, among semisynthetic derivatives, glycyrrhizic acid conjugates with isoleucine and aminodecanoic acid were found as potent anti-DENV-2 inhibitors, with range of IC_50_ between 1.2–1.3 μM [51]; while valine or phenylalanine moieties showed, in vitro, the cytopathic effect 0.50 and 0.18 μM, respectively [52]. Taking into account the fact that the anti-Dengue antiviral activity of OA derivatives is still not known, we have evaluated compounds **5–8** and **23** against all dengue virus serotypes (DENV-1, -2, -3, -4) using the possibilities of cooperation in the framework of the joint project [53].

As follows from the results presented in the Table 2, all studied compounds have demonstrated moderate anti-Dengue virus activity. Among the viruses used, DENV-3 appeared the most resistant to the compounds, while for DENV of other genotypes, the values of CC_50_ exceeded those of IC_50_ two- to five-fold. Compound **6** with ethylpiperazine fragment inhibited 50% of DENV-1 and DENV-2 growth at concentration 67 and 86 µM, correspondingly. Compound **7** was moderately active towards DENV-1 and DENV-2 with 164 and 105 µM, correspondingly. C-azepano-erythrodiol **23** inhibited DENV-2 growth by 50% at concentration 68 µM; while compounds **5** and **8** were inactive regarding all tested DENV serotypes. Our results reveal the OA is a promising scaffold for the synthesis of derivatives with an activity against DENV-1 and 2 types.

## 3. Materials and Methods

### 3.1. Materials

The spectra of synthesized compounds were recorded at the Center for the Collective Use “Chemistry” of the Ufa Institute of Chemistry of the UFRC RAS and RCCU “Agidel” of the UFRC RAS. ^1^H and the ^13^C-NMR spectrum were recorded on a “Bruker AM-500” (Bruker, Billerica, MA, USA, 500 and 125.5 MHz, respectively, δ, ppm, Hz) in CDCl_3_, internal standard tetramethylsilane. The mass spectra were obtained on a liquid chromatograph–mass spectrometer LCMS-2010 EV (Shimadzu, Kyoto, Japan). The melting points were detected on a micro table “Rapido PHMK05” (Nagema, Dresden, Germany). The optical rotations were measured on a polarimeter “Perkin-Elmer 241 MC” (Perkin Elmer, Waltham, MA, USA) in a tube length of 1 dm. Elemental analysis was performed on a Euro EA-3000 CHNS analyzer (Eurovector, Milan, Italy); the main standard is acetanilide. Thin-layer chromatography analyses were performed on Sorbfil plates (Sorbpolimer, Krasnodar, Russian), using the solvent system chloroform–ethyl acetate, 40:1. Substances were detected by 10% H_2_SO_4_ with subsequent heating to 100–120 °C for 2–3 min. The oleanolic acid was purchased in Xi’an Rongsheng Biotechnology Co Ltd. (ISO9001:2008 and GMP certified company). Compounds **5** [26], **13** and **14** [37], **21** [38], **23** [35] were obtained according to the methods described previously.

### 3.2. Synthesis of Compounds (***2***–***4***, ***6***)

A solution of oleanonic acid 1 (0.46 g, 1 mmol) in anhydrous CHCl_3_ (20 mL) and (COCl)_2_ (0.26 mL, 3 mmol) was stirred at room temperature for 2 h, then concentrated to dryness under reduced pressure. A resulting acid chloride was dissolved in anhydrous CHCl_3_ (30 mL) and treated with homopiperazine (0.15 g, 1.5 mmol) (for compound **2**), spermine (0.3 g, 1.5 mmol) (for compound **3**), spermidine (0.24 mL, 1.5 mmol) (for compound **4**) or aminoethylpiperazine (0.2 mL, 1.5 mmol) (for compound **6**) and three drops of Et_3_N. The mixture was stirred at room temperature for 3 h, washed with 5% HCl solution (2 × 100 mL) and H_2_O (100 mL), dried over CaCl_2_, the solvent was removed under reduced pressure. The product was purified by column chromatography with Al_2_O_3_ using CHCl_3_ and a mixture of CHCl_3_–EtOH (100:1) as eluents.

#### 3.2.1. *N*-(3-Oxo-olean-12(13)-en-28-oyl)-homopiperazine Amide (**2**)

Yield 0.44 g (83%); m.p. 174 °C; [α]_D_^20^ + 28 °(c 0.01, CHCl_3_); ^1^H NMR (CDCl_3_, 500.13 MHz,): δ = 7.41 (1H, br.s, CONH), 5.22 (1H, s, H-12), 3.49–3.71 (4H, m, CH_2_), 2.73–3.11 (6H, m, CH_2_), 1.21–2.05 (23H, m, CH, CH_2_, NH), 1.14 (3H, s, H-27), 1.11 (3H, s, H-23), 1.05 (3H, s, H-24), 0.90 (3H, s, H-25), 0.89 (3H, s, H-29), 0.85 (3H, s, H-30), 0.82 (3H, s, H-26); ^13^C NMR (CDCl_3_, 125.76 MHz,): δ = 217.6 (C, C-3), 175.6(C, C-28), 145.1 (C, C-13), 123.2 (CH, C-12), 53.1, 50.5, 50.0, 47.9, 47.6, 47.3, 46.6, 45.5, 45.2, 44.2, 43.9, 42.3, 39.1, 36.4, 34.2, 33.1, 32.1, 30.4(2C), 30.2, 29.9, 29.7, 28.1, 25.7, 24.1, 23.6, 22.6 (2C), 20.3, 16.7, 15.3; EIMS m/z 537.5 [M+H]+ (calcd. 536.85); Anal. Calcd for C_35_H_56_N_2_O_2_: C, 78.31; H, 10.51; N, 5.22. Found: C, 78.24, H 10.42, N, 5.17.

#### 3.2.2. *N*-(3-Oxo-olean-12(13)-en-28-oyl)-spermine Amide (**3**)

Yield 0.33 g (51%); m.p. 184 °C; [α]_D_^20^ − 85° (c 0.01, CHCl_3_); ^1^H NMR (CDCl_3_, 500.13 MHz,): δ = 6.41 (1H, br.s, CONH), 5.31 (1H, s, H-12), 2.21–3.01 (20H, m, CH_2_), 1.21–2.05 (27H, m, CH, CH_2_, NH, NH_2_), 1.15 (3H, s, H-27), 1.12 (3H, s, H-23), 1.08 (3H, s, H-24), 1.05 (3H, s, H-25), 0.92 (3H, s, H-29), 0.89 (3H, s, H-30), 0.78 (3H, s, H-26); ^13^C NMR (CDCl_3_, 125.76 MHz,): δ = 217.8 (C, C-3), 175.7 (C, C-28), 143.9 (C, C-13), 122.0 (CH, C-12), 55.3, 53.0, 47.4, 46.9, 46.6, 46.5, 45.4, 45.3, 44.0, 41.9, 41.8, 41.1, 41.2, 39.3, 39.2, 36.8, 36.4, 34.1, 33.9, 33.1, 32.4, 31.8, 30.7, 29.6, 27.7, 26.4, 25.8, 25.7, 23.6, 23.5, 23.0, 22.6, 21.5, 20.3, 17.0, 16.6; EIMS m/z 639.5 [M+H]+ 20% (calcd. 639.03); Anal. Calcd for C_40_H_70_N_4_O_2_: C, 75.18; H, 11.04; N, 8.77. Found: C, 75.04, H 10.92, N, 8.65.

#### 3.2.3. *N*-(3-Oxo-olean-12(13)-en-28-oyl)-spermidine Amide (**4**)

Yield 0.3 g (52%); m.p. 178–180°C; [α]_D_^20^ − 7° (c 0.01, CHCl_3_); ^1^H NMR (CDCl_3_, 500.13 MHz,): δ = 6.51–6.59 (1H, br.s, CONH), 5.37 (1H, s, H-12), 3.41–2.62 (14H, m, CH_2_), 1.25–2.01 (24H, m, CH, CH_2_, NH, NH_2_), 1.17 (3H, s, H-27), 1.08 (3H, s, H-23), 1.02 (3H, s, H-24), 0.91 (3H, s, H-25), 0.89 (3H, s, H-29), 0.79 (3H, s, H-30), 0.78 (3H, s, H-26); ^13^C NMR (CDCl3, 125.76 MHz,): δ = 217.5 (C, C-3), 178.3 (C, C-28), 144.8 (C, C-13), 122.5 (CH, C-12), 55.2, 47.5, 46.8, 46.7, 46.6, 46.4, 46.3, 42.2, 42.1, 39.4, 39.0, 38.9, 38.3, 36.7, 34.1, 32.6, 31.9, 30.1, 29.4, 27.3, 27.0, 26.4, 26.0, 25.7, 25.6, 23.7, 23.6, 21.5 (2C), 21.3, 19.5, 16.9, 15.1; EIMS m/z 582.5 [M+H]+ (calcd. 581.93); Anal. Calcd for C_37_H_63_N_3_O_2_: C, 76.37; H, 10.91; N, 7.22. Found: C, 76.28, H 10.84, N, 7.11.

#### 3.2.4. *N*-(3-Oxo-olean-12(13)-en-28-oyl)-aminoethylpiperazine Amide (**6**)

Yield 0.38 g (68%); m.p. 175°C; [α]_D_^20^ + 121° (c 0.01, CHCl_3_); ^1^H NMR (CDCl_3_, 500.13 MHz): δ = 6.23 (1H, br.s, CONH), 5.36 (1H, t, ^3^*J* = 3.6, H-12), 3.46 (1H, dq, ^2^*J* = 13.9, ^3^*J*_1′b-2′_ = 6.3, ^3^*J*_1′b-NH_ = 6.3, H_b_-1′), 3.20–3.46 (4H, m, 2CH_2_), 2.83–2.78 (4H, m, 2CH_2_), 2.57–2.46 (6H, m, CH, CH_2_), 1.99–1.19 (21H, m, CH, CH_2_), 1.18 (3H, s, H-27), 1.09 (3H, s, H-23), 1.05 (3H, s, H-24), 1.04 (3H, s, H-25), 0.92 (3H, s, H-29), 0.91 (3H, s, H-30), 0.81 (3H, s, H-26); ^13^C NMR (CDCl_3_, 125.76 MHz): 217.4 (C, C-3), 178.1 (C, C-28), 145.0 (C, C-13), 122.2 (CH, C-12), 56.6, 55.2, 49.5, 47.4, 46.7, 46.6, 46.4, 43.6, 42.3, 42.2, 39.3, 39.0, 36.7, 35.9, 34.2, 34.1, 32.9, 32.7, 31.9, 30.7, 27.3, 26.5, 25.6, 23.8, 23.7, 23.6, 21.5, 19.5, 16.9, 15.1; EIMS m/z 566.5 [M+H]+ (calcd. 565.89); Anal. Calcd for C_36_H_59_N_3_O_2_: C, 76.41; H, 10.51; N, 7.43. Found: C, 76.32, H 10.39, N, 7.34.

### 3.3. Synthesis of Compounds (***7***, ***8***)

To a solution of compound **2** or **3** (0.54 or 0.64 g, 1 mmol) in AcOH (20 mL), phenylhydrazine (0.4 g, 3.5 mmol) was added and refluxed for 18 h, neutralized with 10% HCl solution (100 mL), the precipitate was filtered, washed with H_2_O and 10% NaHCO_3_, and dried in air. The residue was purified by column chromatography on Al_2_O_3_ using CHCl_3_ as an eluent.

#### 3.3.1. *N*-([3,2b]-Indolo-olean-12(13)-en-28-oyl)-homopiperazine Amide (**7**)

Yield 0.39 g (65%); m.p. > 200 °C; [α]_D_^20^ + 44°(c 0.01, CHCl_3_); ^1^H NMR (CDCl_3_, 500.13 MHz,): δ = 7.92(1H, br.s, CONH), 7.34–7.45 (1H, m, H-3″), 7.23–7.31 (1H, m, H-6″), 6.98–7.14 (2H, m, H-4″, H-5″), 5.37 (1H, s, H-12), 2.89–3.19 (4H, m, H-2′, H-7′), 2.69–2.81 (4H, m, H-3′, H-5′), 2.03–2.22 (2H, m, H-6′), 1.21–2.01 (22H, m, CH, CH_2_, NH), 1.14 (3H, s, H-27), 1.11 (3H, s, H-23), 1.05 (3H, s, H-24), 1.01 (3H, s, H-25), 0.93 (3H, s, H-29), 0.89 (3H, s, H-30), 0.78 (3H, s, H-26); ^13^C NMR (CDCl_3_, 125.76 MHz,): δ = 175.9 (C, C-28), 144.6 (C, C-13), 140.9 (C, C-3), 136.2 (C, C_arom_), 128.3 (C, C-1″), 121.8 (CH, C-12), 120.9 (C, C_arom_), 118.8 (C, C_arom_), 117.9 (C, C_arom_), 110.3 (C, C_arom_), 106.9 (C, C-2), 53.3, 47.9, 47.5, 46.5, 46.6, 43.9, 42.2, 39.4, 38.2, 36.8, 34.2, 34.0, 33.1, 32.5, 31.0, 30.4 (2C), 30.1, 29.8, 28.2, 25.8, 24.1, 23.5, 23.4, 23.3, 22.7(2C), 19.4, 17.0, 15.6; EIMS m/z 610.6 [M+H]+ (calcd. 609.94); Anal. Calcd for C_41_H_59_N_3_O: C, 80.74; H, 9.75; N, 6.89. Found: C, 80.67, H 9.63, N, 6.81.

#### 3.3.2. *N*-([3,2b]-Indolo-olean-12(13)-en-28-oyl)-spermine Amide (**8**)

Yield 0.42 g (59%); m.p. 138 °C; [α]_D_^20^ + 27°(c 0.01, EtOH); ^1^H NMR (CDCl_3_, 500.13 MHz,): δ = 7.74 (1H, br s, NH), 7.54–6.97 (4H, m, H_arom_), 5.35 (1H, s, H-12), 3.53–3.30 (2H, m, H-1′), 3.10–1.09 (46H, m, CH, CH_2_), 1.15 (3H, s, H-27), 1.12 (3H, s, H-23), 1.08 (3H, s, H-24), 1.02 (3H, s, H-25), 0.92 (3H, s, H-29), 0.89 (3H, s, H-30), 0.78 (3H, s, H-26); ^13^C NMR (CDCl_3_, 125.76 MHz,): δ = 175.8 (C, C-28), 143.4 (C, C-13), 140.8 (C, C-3), 136.2 (C, C_arom_), 129.8 (C, C_arom_), 122.9 (CH, C-12), 120.9 (C, C_arom_), 118.9 (C, C_arom_), 117.9 (C, C_arom_), 110.3 (C, C_arom_), 106.9 (C, C-2), 53.1, 46.5, 46.3, 42.2, 39.6, 38.1, 37.7, 36.8, 34.3, 34.0, 33.1 (2C), 31.9 (4C), 31.0, 30.8, 29.7, 29.2, 27.5, 27.1, 26.8 (2C), 25.7 (2C), 24.9, 23.7, 23.6, 23.3, 22.7, 19.4, 16.9, 16.8, 15.7; EIMS m/z 712.5 [M+H]+ (calcd. 712.12); Anal. Calcd for C_46_H_73_N_5_O: C, 77.59; H, 10.33; N, 9.83. Found: C, 77.46, H 10.21, N, 9.71.

### 3.4. Synthesis of Compounds (***9***, ***10***)

To a suspension of paraformaldehyde (0.5 g) in dry ethanol (15 mL), benzylamine (0.11 mL, 1.2 mmol) was added and the mixture was stirred under argon atmosphere at room temperature for 1 h, followed by the addition of the ethyl isocyanoacetate (0.11 mL, 1 mmol) and oleanolic **1** (0.45 g, 1 mmol) or 2,3-indolo-oleanolic acid (0.53 g, 1 mmo,). After stirring for 14 days at room temperature and the regular control of the reaction progress by TLC, the solvent was roto-evaporated, the residue dissolved in dichloromethane, the organic layer was washed with 1N HCl and with saturated NaHCO_3_ and with water. The org layer was separated, dried over MgSO_4_ and the solvent evaporated. The residue was purified over SiO_2_ with MDC and 0–5% MeOH to give the UGI product.

#### 3.4.1. Ethyl *N*-Benzyl-*N*-3-oxo-olean-12-en-28-glycylglycinate (**9**)

Yield 0.52 g (75%); m.p. 245–247 °C; [α]_D_^20^ + 45° (c 0.01, CHCl_3_); ^1^H NMR (CDCl_3_, 500.13 MHz): δ = 7.15–7.40 (5H, m, H_arom_), 6.65 (1H, br.s, *N*H), 4.95 (1H, s, H-12), 3.71–4.21 (8H, m, 4CH_2_), 1.20–3.00 (26H, m, CH, CH_2_), 1.47 (3H, s, H-27), 1.21 (3H, s, H-23), 1.11 (3H, s, H-24), 1.07 (3H, s, H-25), 1.03 (3H, s, H-29), 0.92 (3H, s, H-30), 0.87 (3H, s, H-26); ^13^C NMR (CDCl_3_, 125.76 MHz): δ = 217.8 (C, C-3), 178.1 (C, C-28), 170.0 (C, C(O)NH), 169.6 (C, C(O)O), 144.8 (C, C-13), 136.3 (C, H-2′), 128.8 (2CH, C-4′, C-6′), 127.7 (2CH, C-3′, C-7′), 127.2 (CH, C-5′), 121.4 (CH, C-12), 61.4 (CH_2_, C-12′), 55.4 (CH_2_, C-8′), 52.9, 52.3 (CH_2_, C-1′), 51.1, 48.1, 47.5, 46.9, 46.8, 43.6, 42.0, 41.0 (CH_2_, C-10′), 39.2, 39.1. 36.8, 34.2, 34.1, 32.9, 32.4, 30.3, 30.3, 28.1, 26.4, 25.7, 24.1, 23.5, 21.5, 19.6, 16.9, 15.0, 14.2. EIMS m/z 687.6 [M+H]+ (calcd. 686.98); Anal. Calcd for C_43_H_62_N_2_O_5_: C, 75.18; H, 9.10; N, 4.08. Found: C, 75.27; H, 9.21; N, 4.15.

#### 3.4.2. Ethyl *N*-Benzyl-*N*-2,3-indolo-olean-12-en-28-glycylglycinate (**10**)

Yield 0.54 g (71%); m.p. 197–199 °C; [α]_D_^20^ + 21° (c 0.01, CHCl_3_); ^1^H NMR (CDCl_3_, 500.13 MHz): δ = 7.90 (1H, br.s, *N*H), 7.15–7.40 (9H, m, H_arom_), 6.68 (1H, br.s, *N*H), 5.00 (1H, s, H-12), 3.90–4.25 (8H, m, 4CH_2_), 1.40–3.10 (24H, m, CH, CH_2_), 1.32 (3H, s, H-27), 1.30 (3H, s, H-23), 1.28 (3H, s, H-24), 1.25 (3H, s, H-25), 1.21 (3H, s, H-29), 0.98 (3H, s, H-30), 0.93 (3H, s, H-26); ^13^C NMR (CDCl_3_, 125.76 MHz): δ = 178.2 (C, C-28), 171.0 (C, C(O)NH), 169.6 (C, C(O)O), 144.5 (C, C-13), 140.8 (C, C-3), 136.3 (C, H-2′), 136.1 (C, C-14′), 128.7 (2CH, C-4′, C-6′), 128.3 (C, C-19′), 127.6 (2CH, C-3′, C-7′), 127.3 (CH, C-5′), 121.9 (CH, C-12), 120.9 (C, C-16′), 118.8 (C, C-17′), 117.9 (C, C-18′), 110.3 (C, C-15′), 106.9 (C, C-2), 61.4 (CH_2_, C-12′), 55.4 (CH_2_, C-8′), 52.3 (CH_2_, C-1′), 51.1, 48.2, 46.8, 46.4, 42.1, 41.2 (CH_2_, C-10′), 39.4, 38.2, 36.8, 34.1, 34.0, 32.9, 32.4, 30.9, 30.4, 28.2, 25.7, 24.1, 23.4, 23.3, 23.1, 19.4, 17.1, 16.9, 15.6, 14.2, 14.1. EIMS m/z 760.6 [M+H]+ (calcd. 760.08); Anal. Calcd for C_49_H_65_N_3_O_4_: C, 77.43; H, 8.62; N, 5.53. Found: C, 77.52; H, 8.55; N, 5.64.

### 3.5. Synthesis of Compound (***11***)

The solution of compound **2** (0.54 g, 1 mmol) in DMF (20 mL), chloroacetonitrile (0.08 mL, 1.26 mmol) and K_2_CO_3_ (135 mg, 0.98 mmol) were added and stirred at 60 °C for 1 h. The mixture was poured into ice-cold water and the precipitate was filtered. The residue was dissolved in EtOAc (100 mL), washed with water (50 mL) and brine (50 mL × 2), dried over Na_2_SO_4_, filtered, and concentrated.

#### *N*-(3-Oxo-olean-12(13)-en-28-oyl)-4′-cyanomethyl-homopiperazine Amide (**11**)

Yield 0.47 g (82%); m.p. 191 °C; [α]_D_^20^ − 29° (c 0.01, CHCl_3_); ^1^H NMR (CDCl_3_, 500.13 MHz,): δ = 5.23 (1H, s, H-12), 4.39–4.61 (2H, m, CH_2_CN), 3.76–3.42 (6H, m, CH_2_), 2.55–3.12 (4H, m, CH_2_), 2.81–1.10 (23H, m, CH, CH_2_), 1.11 (3H, s, H-27), 0.90 (3H, s, H-23), 0.88 (3H, s, H-24), 0.87 (3H, s, H-25), 0.82 (3H, s, H-29), 0.81 (3H, s, H-30), 0.72 (3H, s, H-26); ^13^C NMR (CDCl_3_, 125.76 MHz,): δ = 217.7 (C, C-3), 175.7 (C, C-28), 144.8 (C, C-13), 121.3 (CH, C-12), 115.2 (C, CN), 55.3, 47.7, 47.6, 47.3, 46.9. 46.4, 43.7, 41.9, 39.1, 38.0, 37.7, 36.9, 34.0, 33.0, 32.7, 30.4, 30.3, 29.9, 27.9, 27.9, 25.9 (2C), 24.1, 23.5, 23.3, 22.5, 21.3 (2C), 18.2, 16.9, 16.6, 15.4; EIMS m/z 576.4 [M+H]+ (calcd. 575.88); Anal. Calcd for C_37_H_57_N_3_O_2_: C, 77.17; H, 9.98; N, 7.30. Found: C, 77.02, H 9.86, N, 7.24.

### 3.6. Synthesis of Compound (***12***)

To a solution of compound **11** (0.57 g, 1 mmol) in DMF (20 mL), NaN_3_ (0.2 g, 3 mmol) and NH_4_Cl (0.1 g, 2 mmol) were added and refluxed for 8 h, neutralized with 5% HCl solution (100 mL), the precipitate was filtered, washed, dried on air and recrystallized from EtOH.

#### *N*-(3-Oxo-olean-12(13)-en-28-oyl)-4′-tetrazolomethyl-homopiperazine Amide (**12**)

Yield 0.47 g (82%); 166–168 °C; [α]_D_^20^ + 18° (c 0.01, CHCl_3_); ^1^H NMR (CDCl_3_, 500.13 MHz,): δ = 5.23 (1H, s, H-12), 4.45 (2H, t, *J* = 8, CH_2_CN), 3.75–3.20 (6H, m, 3CH_2_), 3.00–1.10 (28H, m, CH, CH_2_), 1.12 (3H, s, H-27), 0.91 (3H, s, H-23), 0.87 (3H, s, H-24), 0.86 (3H, s, H-25), 0.82 (3H, s, H-29), 0.81 (3H, s, H-30), 0.72 (3H, s, H-26); ^13^C NMR (CDCl_3_, 125.76 MHz,): δ = 217.4 (C, C-3), 171.1 (C, C-28), 162.7 (C, C-9′), 144.7 (C, C-13), 121.4 (CH, C-12), 55.4, 47.8, 47.7, 47.5 (2C), 46.7, 42.0, 39.1, 38.1, 37.7, 37.0, 34.1 (2C), 33.0, 32.9, 30.3, 30.1, 28.0, 25.2 (3C), 24.0, 23.5, 23.4, 21.3 (2C), 18.3, 17.2, 17.1, 16.7, 15.4, 15.3; EIMS m/z 619.5 [M+H]+ (calcd. 618.91); Anal. Calcd for C_37_H_58_N_6_O_2_: C, 71.73; H, 9.31; N, 13.46. Found: C, 71.80, H 9.45, N, 13.58.

### 3.7. Synthesis of Compounds (***15***, ***16***)

A solution of compound **13** or **14** (0.54 g, 1 mmol) in isopropanol (20 mL) was treated with NaBH_4_ (50 mg, 1.3 mmol), stirred for 2 h, then diluted with 10% HCl (30 mL). The residue was filtered off, washed with H_2_O, dried, and recrystallized from EtOH.

#### 3.7.1. 3*β*-Hydroxy-2-(3-pyridinoylidene)-olean-12-en-28-oic Acid (**15**)

Yield 0.45 g (83%); m.p. 201–203 °C; [α]_D_^20^ + 45° (c 0.01, CHCl_3_); ^1^H NMR (CDCl_3_, 500.13 MHz,): δ = 11.0 (1H, br. s, COOH), 8.45 (1H, br.s, H_arom_), 8.36 (1H, br. s, H_arom_), 7.51 (1H, d, *J* = 7.8 Hz, H_arom_), 7.22–7.32 (1H, m, H_arom_), 6.67 (1H, br. s, H-1′), 5.18 (1H, s, H-12), 3.87 (1H, m, H-3), 2.85 (1H, d, *J* = 10.1 Hz, H-1a), 2.64 (1H, d, *J* = 12.8 Hz, H-1b), 1.16–1.99 (20H, m, CH, CH_2_), 1.13 (3H, s, H-27), 1.11 (3H, s, H-23), 0.94 (3H, s, H-24), 0.89 (3H, s, H-25), 0.72 (3H, s, H-29), 0.62 (3H, s, H-30), 0.59 (3H, s, H-26); ^13^C NMR (CDCl_3_, 125.76 MHz,): δ = 181.9 (C, C-28), 148.3 (C, C-2), 145.4 (C, C-13), 144.1, 143.9, 137.4, 134.7, 123.4 (2C), 121.9 (CH, C-12), 80.8 (CH, C-3), 55.6, 46.9, 46.3, 46.0, 42.2, 41.9, 41.4, 40.6, 39.5, 33.9, 33.2, 32.4, 30.8, 29.0, 28.7, 27.8, 26.1, 23.9, 23.8, 23.3, 22.8, 18.4, 16.7, 15.6, 15.5; EIMS m/z 546.4 [M+H]+(calcd. 545.81); Anal. Calcd for C_36_H_51_NO_3_: C, 79.22; H, 9.42; N, 2.57. Found: C, 79.12; H, 9.36; N, 2.43.

#### 3.7.2. 3*β*-Hydroxy-2-(4-pyridinoylidene)-olean-12-en-28-oic Acid (**16**)

Yield 0.48 g (88%); m.p. °C; 187–189 °C; [α]_D_^20^ + 15° (c 0.01, CHCl_3_); ^1^H NMR (CDCl_3_, 500.13 MHz,): δ = 11.0 (1H, br. s, COOH), 8.49 (2H, d, *J* = 4.4 Hz, H_arom_), 7.27 (2H, d, *J* = 4.9 Hz, H_arom_), 7.13 (1H, d, *J* = 6.0 Hz, H-1′), 5.24 (1H, s, H-12), 3.88 (1H, s, H-3), 2.78–2.93 (2H, m, H-1), 1.22–2.01 (20H, m, CH, CH_2_), 1.13 (3H, s, H-27), 1.11 (3H, s, H-23), 0.92 (3H, s, H-24), 0.88 (3H, s, H-25), 0.73 (3H, s, H-29), 0.71 (3H, s, H-30), 0.69 (3H, s, H-26); ^13^C NMR (CDCl_3_, 125.76 MHz,): δ = 182.4 (C, C-28), 148.3 (C, C-2), 147.4 (C, C-13), 145.3 (2C), 137.1, 133.7, 121.9 (CH, C-12), 120.4, 80.9 (C, C-3), 55.7, 55.1, 47.0, 46.3, 46.0, 42.2, 41.8, 41.3, 40.6, 39.5, 33.9, 33.1, 30.8, 29.0, 28.7, 27.8, 25.9, 23.9, 23.7, 23.4, 22.9, 18.5, 16.9, 15.8, 15.4; EIMS m/z 546.4 [M+H]+ (calcd. 545.81); Anal. Calcd for C_36_H_51_NO_3_: C, 79.22; H, 9.42; N, 2.57. Found: C, 79.14; H, 9.31; N, 2.40.

### 3.8. Synthesis of Compounds (**17**–**20**)

A solution of compounds **13** or **14** (0.54 g, 1 mmol) in anhydrous CHCl_3_ (20 mL) and (COCl)_2_ (0.26 mL, 3 mmol) was stirred at room temperature for 2 h, then concentrated to dryness under reduced pressure. A residue of acid chloride derivative was dissolved in anhydrous CHCl_3_ (30 mL) and treated with homopiperazine (0.15 g, 1.5 mmol) (for compounds **17** or **19**) or spermine (0.3 g, 1.5 mmol,) (for compounds **18** or **20**) and three drops of Et_3_N. The mixture was stirred at room temperature for 2.5 h, washed with 5% HCl solution (2 × 100 mL) and H_2_O (100 mL), and dried over CaCl_2_. The solvent was removed under reduced pressure. The product was purified by column chromatography with Al_2_O_3_ using CHCl_3_ and a mixture of CHCl_3_–EtOH (100:1) as eluents.

#### 3.8.1. *N*-(2-{3-Pyridinoylidene}-3-oxo-olean-12(13)-en-28-oyl)-homopiperazine Amide (**17**)

Yield 0.43 g (69%); m.p. 194.3 °C; [α]_D_^20^ − 96°(c 0.01, CHCl_3_); ^1^H NMR (CDCl_3_, 500.13 MHz,): δ = 8.62 (1H, br. s, H_arom_), 8.54 (1H, d, *J* = 4.1 Hz, H_arom_), 7.68 (1H, d, *J* = 7.8 Hz, H_arom_), 7.41 (1H, br. s, H_-_1′), 7.19–7.34 (1H, m, H_arom_), 5.24 (1H, s, H-12), 3.49–3.78 (5H, m, CH_2_, NH), 2.72–3.15 (6H, m, CH_2_), 1.27–2.18 (21H, m, CH, CH_2_), 1.18 (3H, s, H-27), 1.11 (3H, s, H-23), 1.08 (3H, s, H-24), 0.91 (3H, s, H-25), 0.89 (3H, s, H-29), 0.83 (3H, s, H-30), 0.78 (3H, s, H-26); ^13^C NMR (CDCl_3_, 125.76 MHz,): δ = 207.4 (C, C-3), 175.6 (C, C-28), 151.4 (C, C-20), 149.1 (C, C-2), 146.7, 145.1 (C, C-13), 136.7, 133.4, 131.8, 123.2 (CH, C-12), 121.0, 53.1, 50.5, 50.0, 47.9, 47.6, 47.3, 46.6, 45.5, 45.2, 44.2, 43.9, 42.3, 39,1, 36.4, 34.2, 33.1, 32.1, 30.4 (2C), 30.2, 29.9, 29.7, 28.1, 25.7, 24.1, 23.6 (2C), 22.6, 16.7, 15.3; EIMS m/z 626.5 [M+H]+ (calcd. 625.94); Anal. Calcd for C_41_H_59_N_3_O_2_: C, 78.67; H, 9.50; N, 6.71. Found: C, 78.54; H, 9.44; N, 6.63.

#### 3.8.2. *N*-(2-{3-Pyridinoylidene}-3-oxo-olean-12(13)-en-28-oyl)-spermine Amide (**18**)

Yield 0.38 g (52%); m.p. > 200 °C; [α]_D_^20^ − 22° (c 0.01, CHCl_3_); ^1^H NMR (CDCl_3_, 500.13 MHz,): δ = 8.63 (1H, br. s, H_arom_), 8.52 (1H, br. s, H_arom_), 7.72 (1H, d, *J* = 7.3 Hz, H_arom_), 7.43 (1H, br. s, H_-_1′), 7.28–7.39 (1H, m, H_arom_), 5.42 (1H, s, H-12), 3.58–2.34 (25H, m, CH_2_, NH, NH_2_), 1.28–2.18 (21H, m, CH, CH_2_), 1.18 (3H, s, H-27), 1.11 (3H, s, H-23), 1.08 (3H, s, H-24), 0.91 (3H, s, H-25), 0.89 (3H, s, H-29), 0.81 (3H, s, H-30), 0.78 (3H, s, H-26); ^13^C NMR (CDCl_3_, 125.76 MHz,): δ = 207.3 (C, C-3), 178.7 (C, C-28), 151.0 (C, C-2), 149.7, 149.0, 144.5 (C, C-13), 136.9, 135.6, 133.5, 123.4, 123.1 (CH, C-12), 55.5, 52.9, 49.0, 47.7, 46.5, 45.3, 44.8, 44.1, 42.3, 42.2, 42.0, 41.9, 39.4, 38.6, 37.1, 36.3, 34.2, 33.0, 32.9, 32.8, 31.9, 31.6, 30.7, 29.7, 29.6, 29.4, 27.3, 26.5, 25.9, 25.7, 23.6, 20.8, 20.3, 16.3, 15.7; EIMS m/z 728.6 [M+H]+ (calcd. 728.12); Anal. Calcd for C_46_H_73_N_5_O_2_: C, 75.88; H, 10.11; N, 9.62. Found: C, 75.71; H, 10.03; N, 9.54.

#### 3.8.3. *N*-(2-{4-Pyridinoylidene}-3-oxo-olean-12(13)-en-28-oyl)-homopiperazine Amide (**19**)

Yield 0.41 g (66%); m.p. > 200 °C; [α]_D_^20^ − 23° (c 0.01, CHCl_3_); ^1^H NMR (CDCl_3_, 500.13 MHz,): δ = 8.63 (2H, d, *J* = 4.9 Hz, H_arom_), 7.25–7.29 (1H, m, H_arom_), 7.23 (1H, d, *J* = 4.9 Hz, H_arom_), 7.12 (1H, br.s, H-1′), 5.25 (1H, s, H-12), 4.01–4.38 (5H, m, CH_2_, NH), 2.78–3.26 (6H, m, CH_2_), 1.18–2.16 (21H, m, CH, CH_2_), 1.14 (3H, s, H-27), 1.12 (3H, s, H-23), 1.09 (3H, s, H-24), 1.01 (3H, s, H-25), 0.93 (3H, s, H-29), 0.86 (3H, s, H-30), 0.81 (3H, s, H-26); ^13^C NMR (CDCl_3_, 125.76 MHz,): δ = 207.4 (C, C-3), 179.1 (C, C-28), 150.3 (C, C-2), 149.7, 143.6 (C, C-13), 138.1, 133.9, 124.2, 122.8 (CH, C-12), 121.9, 120.8, 54.0, 53.1, 47.9, 47.7, 46.8, 46.2, 45.9, 45.8, 45.5, 45.3, 44.0, 43.4, 42.3, 38.9, 38.0, 36.4, 34.3, 32.9, 32.0, 30.3, 27.8, 25.8, 23.6, 23.1, 22.6, 21.8, 20.6, 20.3, 19.5, 16.7; EIMS m/z 626.4 [M+H]+ (calcd. 625.94); Anal. Calcd for C_41_H_59_N_3_O_2_: C, 78.67; H, 9.50; N, 6.71. Found: C, 78.53; H, 9.38; N, 6.62.

#### 3.8.4. *N*-(2-{4-Pyridinoylidene}-3-oxo-olean-12(13)-en-28-oyl)-spermine Amide (**20**)

Yield 0.40 g (56%); m.p. 137 °C; [α]_D_^20^ − 13°(c 0.01, CHCl_3_); ^1^H NMR (CDCl_3_, 500.13 MHz,): δ = 8.63 (1H, br.s, H_arom_), 7.11–7.33 (3H, m, H_arom_), 6.92 (1H, br.s, H-1′), 5.39 (1H, s, H-12), 2.47–3.53 (25H, m, CH_2_, NH, NH_2_), 1.18–2.12 (21H, m, CH, CH_2_), 1.16 (3H, s, H-27), 1.13 (3H, s, H-23), 1.11 (3H, s, H-24), 0.91 (3H, s, H-25), 0.89 (3H, s, H-29), 0.83 (3H, s, H-30), 0.78 (3H, s, H-26); ^13^C NMR (CDCl_3_, 125.76 MHz,): δ = 207.2 (C, C-3), 179.8 (C, C-28), 149.8 (C, C-2), 148.7, 144.5 (C, C-13), 143.6, 142.1, 133.9, 124.1, 123.2, 122.4 (CH, C-12), 53.0, 49.3, 47.1, 46.4, 45.4, 45.3, 43.9, 42.2, 42.0, 41.7, 39.4, 39.3, 38.8, 36.3, 35.7, 34.1, 33.2, 33.0 (2C), 29.7, 29.5, 27.4, 26.1 (2C), 25.8, 25.6 (2C), 23.7, 23,5, 23.0, 22.7, 20.7, 17.8, 16.7, 15.3; EIMS m/z 728.5 [M+H]+ (calcd. 728.12); Anal. Calcd for C_46_H_73_N_5_O_2_: C, 75.88; H, 10.11; N, 9.62. Found: C, 75.76; H, 10.04; N, 9.51.

### 3.9. Synthesis of Compound (***22***)

To a solution of compound **21** (0.62 g, 1 mmol) in anhydrous CH_2_Cl_2_ (20 mL), 1,3-dicyclohexylcarbodiimide (DCC) (0.4 g, 1.5 mmol) and dimethylaminopyridine (DMAP) (0.1 g; 1 mmol) at 0 °C were added, then, after 10 min, phthalic anhydride (0.3 g, 2 mmol) was added and the mixture was stirred at room temperature for 12 h, the precipitate was filtered, the solution was diluted with H_2_O, and extracted with CHCl_3_ (3 × 20 mL). The combined organic layer was washed with 10% NaHCO_3_ and brine, then dried over CaCl_2_. The solvent was removed under reduced pressure. The product was purified by column chromatography on Al_2_O_3_ using petroleum ester—EtOAc (70:1, 40:1, 20:1, 10:1, 5:1) as eluent.

#### Methyl 3*β*-*N*-phthalyl-olean-12(13)-en-28-oate (**22**)

Yield 0.37 g (89%); m.p. 240 °C; [α]_D_^20^ + 65° (c 0.01, CHCl_3_); ^1^H NMR (CDCl_3_, 500.13 MHz,): δ = 9.18 (1H, br.s, OH), 7.61–7.90 (5H, m, H_arom_, NH), 5.51 (1H, br.s, H-12), 3.01 (3H, s, OCH_3_), 1.25–2.34 (24H, m, CH, CH_2_), 0.69 (3H, s, H-27), 0.71 (3H, s, H-23), 0.93 (3H, s, H-24), 0.96 (3H, s, H-25), 1.10 (3H, s, H-29), 1.12(3H, s, H-30), 1.15 (3H, s, H-26); ^13^C NMR (CDCl3, 125.76 MHz): δ = 179.4 (C, C-28), 169.5 (C, CONH), 169.2 (C, COOH), 146.1 (C, C-13), 134.0, 133.9, 133.8, 123.2 (CH, C-12), 123.1, 123.0, 61.1 (CH, C-3), 57.1, 55.7, 52.7, 50.7, 49.6, 43.9, 41.3, 40.6, 39.8, 37.8, 34.3, 33.2, 32.9, 31.1, 29.3, 28.9, 27.0, 26.7, 23.6, 23.6, 22.9, 21.6, 21.3, 20.1, 18.9, 17.9, 17.2; EIMS m/z 618.4 [M]+ 100% (calcd. 617.87); Anal. Calcd for C_39_H_55_NO_5_: C, 75.81; H, 8.97; N, 2.27. Found: C, 75.76; H, 8.74; N, 2.19.

### 3.10. Biological Activity

Viruses and cells. The Influenza virus A/Puerto Rico/8/34 (H1N1) was obtained from the collection of viruses of the St. Petersburg Pasteur Institute. Before the experiment, the virus was propagated in the allantoic cavity of 10 to 12 days old chicken embryos for 48 h, at 36 °C. The infectious titer of the virus was determined in Madin-Darby Canine Kidney (MDCK) cells (ATCC-CCL-34) grown in 96-well plates in alpha-MEM medium with 10% fetal bovine serum.

#### 3.10.1. Cytotoxicity Assay

The MDCK cells were seeded onto 96-well culture plates (104 cells per well) and incubated at 36 °C in 5% CO_2_ until continuous monolayer formation. To assess the toxicity of the compounds, a series of their 3-fold dilutions, at concentrations of 300 to 3.7 μg/mL in Eagle’s Minimal Essential Medium (MEM), were prepared. The dilutions were added to the wells of the plates. The cells were incubated for 72 h at 36 °C in a CO_2_ incubator under 5% CO2. Further, a microtetrazolium (MTT) assay was performed on the 96-well plates. The cells were washed 2 times with saline (0.9% NaCl), and 100 μL/well of MTT solution [3-(4,5-dimethylthiazol-2-yl)-2,5-diphenyltetrazolium bromide] at a concentration of 0.5 μg/mL in MEM was added. The plates were incubated for 1 h at 36 °C, the liquid was removed, and dimethylsulfoxide (DMSO) (0.1 mL per well) was added. The optical density (OD) of the cells was measured on a Thermo Multiskan FC spectrophotometer (Thermo Fisher Scientific, Waltham, MA, USA) at a wavelength of 540 nm. Based on the obtained data, the CC_50_, the concentration of the compound that destroys 50% of the cells in the culture, was calculated for each specimen.

#### 3.10.2. CPE Reduction Assay

The compounds in appropriate concentrations were added to the MDCK cells (0.1 mL per well). The MDCK cells were further infected with A/Puerto Rico/8/34 (H1N1) influenza virus (m.o.i 0.01). The plates were incubated for 72 h at 36 °C at 5% CO_2_. Next, cell viability was assessed by the MTT test, as described above. The cytoprotective activity of the compounds was considered as their ability to increase the values of the OD compared to the control wells (with virus only; no drugs). Based on the obtained results, the IC50 values, i.e., the concentration of compounds that results in 50% cell protection, were calculated using the GraphPad Prism software. The IC_50_ values in μg/mL were then calculated into micromoles. For each compound, the value of the selectivity index (SI) was calculated as a ratio of CC_50_ to IC_50_.

### 3.11. Dengue Virus Propagation

The dengue viruses used in this study were DENV-1:VN/2017/D7709, DENV-2: 00St22A, DENV-3: SMLC50, DENV-4: SLMC318. The virus strains were supplied by the Vietnam National Institute of Hygiene and Epidemiology. The four above serotypes of the Dengue virus were propagated in BHK-21 cell lines (provided by Nagasaki University, Japan) in Eagle’s Minimum Essential Medium (EMEM; Gibco Thermo Fischer Scientific, Waltham, MA, USA) with 2% Fetal Bovine Serum (FBS; Sigma Aldrich, St. Louis, MO, USA) at 37 °C under 5% CO_2_ atmosphere for 5–7 days. The BHK-21 cells were cultured using 10% FBS EMEM medium.

#### 3.11.1. Plaque Reduction Neutralization Test (PRNT)

##### Preparation of Cells in 24-Well-Plates

EMEM (1 mL) containing FBS (2%) was added into each of 24-well plates (with a concentration of 0.5.0 × 10^4^ –1.0 × 10^4^ cells/well). The well plates were shaken to induce the formation of identical cell layers. Afterwards, the wells were incubated at 37 °C under 5% CO_2_ atmosphere overnight until the cell concentration reached at 70–90%. In case this concentration was not yet reached, the incubation was allowed to continue for a further 1 to 2 days.

##### Plaque Reduction Neutralization Test (PRNT)

The compounds **4**–**8** and **23** were totally dissolved in EMEM with a concentration of 500 μg/mL, which was serially diluted with step 2 with EMEM containing 2% FBS; the solutions of compounds were kept at 2–8 °C. The viruses were used at the dose of 2.5 × 10^3^ PFU/mL in EMEM containing 2% FBS. The virus (100 μL) was added to all the diluted samples. The virus-compounds mixtures were incubated at 37 °C under 5% CO_2_ atmosphere for 60 min; afterwards, the medium in all wells of the plates was removed. The virus-compounds mixture (50 μL) was added into the wells, according to the designed experimental test concentration/dilution.

The viral medium was used as the negative control. EMEM (2 mL) containing methyl cellulose (1%) was added and re-incubated at 37 °C under 5% CO_2_ atmosphere for 4–6 days. The typical DENV-1 and DENV-4-induced CPE appeared after 4 days of incubation, while the durations for DENV-2 and DENV-3 were 5 and 6 days, respectively. These times of incubation allowed the formation of the plaques to be visually observable and countable. The plaques can be observed under a microscope or by eyes. The cells were fixed with formalin (3.7%) for 1 h at room temperature, washed with water, and stained with crystal violet (0.25%) for 1 h at room temperature. Finally, the plaques were counted.

### 3.12. Cytotoxic Assay

The cytotoxic evaluation of the extracts was carried out against the BHK-21 cells [54,55]. Under cultural conditions of EMEM with FBS (10%), the assaying cells were grown in 96-well bottom-transparent plates at a density of 6 × 10^4^ cells/mL per well. The compounds that had been dissolved in dimethyl sulfoxide (DMSO) with six different concentrations (500, 250, 125, 62.5, 31.25, 15.625 μg/mL) were added into each well; each concentration was used in triplicate. After incubation at 37 °C under 5% CO_2_ atmosphere for 48 h, MTT (0.5 mg/mL) was added into each well; afterwards, 4 h incubation was carried out. The liquid in the wells was removed, and DMSO (100 mL) was added to each well. The optical absorbance was recorded on a microplate reader at λ = 570 nm (Genious Tecan, Grödig, Australia). The optical density in the wells was plotted against the logarithm of compound concentration, and 50% cytotoxic concentration (CC_50_), the compound concentration decreasing the optical density twice comparing to control wells, was calculated using the appropriate software (Table Curve 2Dv4.USA).

### 3.13. Statistics

All of the in vitro experiments were repeated three times. The results of the “dose-response” experiments were analyzed using GraphPad Prism 6.01. The values of CC_50_ and IC_50_ were calculated as the mean of three independent values and represented as mean ± SD. The selectivity index for each compound was calculated as the ratio of CC_50_ to IC_50_.

## 4. Conclusions

A series of sixteen A-ring modified (2,3-indolo-, 2-benzylidene) oleanonic acid derivatives holding some cyclic amines, linear polyamines and benzylaminocarboxamides at C28 were synthesized and screened for antiviral activity against influenza A (H1N1) and Dengue virus serotypes of DENV. The tested OA-derivatives were of relatively low cytotoxicity and antiviral activity at the level or higher of the reference drug rimantadine. Among them, 28-homopiperazine **2** and 3-*N*-phthalyl **22** amides of oleanonic acid demonstrated high potency, with a selectivity index of SI 27 (IC_50_ 21 μM) and 42 (IC_50_ 12 μM), against flu A. Oleanonic acid аminoethylpiperazine amide **6** and C-azepano-erythrodiol **23** appeared to be the most effective against the DENV-1 (IC_50′_s 67 and 107 μM) and -2 (IC_50′_s 86 and 68 μM correspondingly) serotypes. Among the viruses used, DENV-3 appeared the most resistant to the compounds, while the other genotypes of DENV demonstrated values of CC_50_ that exceeded those of IC_50_ by two- to five-fold. These data demonstrate that the OA scaffold is very potent for the generation of new antiviral compounds among plant metabolites. Further studies on this topic are in progress in our international collaborative group.

## Figures and Tables

**Table 1:** The antiviral activity of compounds **2**, **5**, **6**, **8–11**, and **22** against influenza virus A/Puerto Rico/8/34 (H1N1).

Compound ^a^	CC_50_, μM ^b^	IC_50_, μM ^c^	SI ^d^
**2**	>560	21 ± 3	>27
**5**	50 ± 4	>20	>2
**6**	442 ± 31	82 ± 10	5
**8**	>140	27 ± 4	>5
**9**	>437	41 ± 5	>11
**10**	121 ± 9	39 ± 4	3
**11**	>522	45 ± 6	>12
**21**	18 [40]	7.9 [40]	2 [40]
**22**	>486	12 ± 2	>42
Rimantadine	344 ± 19	61 ± 8	6
Oseltamivir carboxylate	>200	0.3 ± 0.01	>667

^a^ Compounds that are not shown in this table were cytotoxic on normal cell line MDCK; ^b^ CC_50_—50% cytotoxic concentration; ^c^ IC_50_—50% inhibitory concentration; ^d^ SI—Selectivity index is defined as: SI CC_50_/IC_50_. Bold values represent best results (SI value of 10 or higher).

**Table 2:** The anti-Dengue activity of compounds **5**–**8** and **23**.

Compound	CC_50_, μM ^a^	PRNT_50_, μM ^b^			
		DENV-1	DENV-2	DENV-3	DENV-4
**5**	>463	220 ± 14	372 ± 15	452 ± 26	433 ± 31
**6**	111 ± 8	67 ± 4	86 ± 5	>111	110 ± 8
**7**	>410	164 ± 11	105 ± 7	>410	371 ± 16
**8**	>351	336 ± 22	347 ± 21	>351	336 ± 28
**23**	>544	107 ± 7	68 ± 3	228 ± 16	108 ± 5

^a^ 50% cytotoxic concentration; ^b^ Concentration that reduces plaque number by 50%.

## Data Availability

The data presented in this study are available on request from the corresponding authors.

## Supplementary Materials

The following supporting information can be downloaded at: https://www.mdpi.com/article/10.3390/molecules27238499/s1, Figures S1–S34 ^1^H, ^13^C{1H} NMR and mass spectra of compounds **2**–**4**, **6**–**12**, **15**–**20** and **22**.

## Sample Availability

Samples of the compounds **1–23** are available from the authors.

## References

[1] Kvasnica M., Urban M.N., Dickinson J., Sarek J. (2015). Pentacyclic triterpenoids with nitrogen- and sulfur-containing heterocycles: Synthesis and medicinal significance. Nat. Prod. Rep..

[2] Salvador J.A.R., Daniela A.S.L., Alho P.S., Gonçalves B.M.F., Valdeira A.S., Mendes V.I.S., Jing Y. (2014). Chapter 2—Highlights of pentacyclic triterpenoids in the cancer settings. Studies in Nat. Prod. Chem..

[3] Ghiulai R., Rosca O.J., Antal D.S., Mioc M., Mioc A., Racoviceanu R., Macaşoi I., Olario T., Dehelean C., Creţu O.M. (2020). Tetracyclic and pentacyclic triterpenes with high therapeutic efficiency in wound healing approaches. Molecules.

[4] Salvador J.A.R., Leal A.S., Valdeira A.S., Goncalves B.M.F., Alho D.P.S., Figueiredo S.A.C., Silvestre S.M., Mendes V.I.S. (2017). Oleanane-, ursane-, and quinone methide friedelane-type triterpenoid derivatives: Recent advances in cancer treatment. Eur. J. Med. Chem..

[5] Hoenke S., Christoph M., Friedrich S., Heise N., Brandes B., Deigner H.-P., Al-Harrasi A., Csuk R. (2021). The presence of a cyclohexyldiamine moiety confers cytotoxicity to pentacyclic triterpenoids. Molecules..

[6] Pai S.R., Upadhya V., Hegde H.V., Joshi R.K., Kholkute S.D. (2016). Determination of betulinic acid, oleanolic acid and ursolic acid from *Achyranthes aspera* L. using RP-UFLC-DAD analysis and evaluation of various parameters for their optimum yield. Indian J. Exp. Biol..

[7] Javed S., Oise I.E., Nahar L., Ismail F.M.D., Mahmood Z., Sarker S.D. (2016). Isolation, Identification and Antiproliferative Activity of Triterpenes from the Genus *Monotheca* A. DC. Rec. Nat. Prod..

[8] Vetal M.D., Chavan R.S., Rathod V.K. (2014). Microwave assisted extraction of ursolic acid and oleanolic acid from *Ocimum sanctum*. Biotechnol. Bioprocess Eng..

[9] Wang W., Li Y., Li Y., Sun D., Li H., Chen L. (2022). Recent Progress in Oleanolic Acid: Structural Modification and Biological Activity. Curr. Top. Med. Chem..

[10] Yu Z., Sun W., Peng W., Yu R., Li G., Jiang T. (2016). Pharmacokineti Partially in vitro and in vivo of two novel prodrugs of oleanolic acid in rats and its hepatoprotective effects against liver injury induced by CCl_4_. Mol. Pharm..

[11] Gupta N. (2022). A Review on Recent Developments in the Anticancer Potential of Oleanolic Acid and its Analogs (2017–2020). Mini Rev. Med. Chem..

[12] Khwaza V., Oyedeji O., Aderibigbe B. (2018). Antiviral Activities of Oleanolic Acid and Its Analogues. Molecules..

[13] Kaushik S., Dar L., Kaushik S., Yadav J.P. (2021). Anti-dengue activity of super critical extract and isolated oleanolic acid of *Leucas cephalotes* using in vitro and in silico approach. BMC Complement. Med. Ther..

[14] Li W., Yang F., Meng L., Sun J., Su Y., Shao L., Zhou D., Yu F. (2019). Synthesis, Structure Activity Relationship and Anti-influenza A Virus Evaluation of Oleanolic Acid-Linear Amino Derivatives. Chem. Pharm. Bull..

[15] Li H., Cheng C., Li S., Wu Y., Liu Z., Liu M., Chen J., Zhong Q., Zhang X., Liu S. (2021). Discovery and structural optimization of 3-O-b-chacotriosyl oleanane-type triterpenoids as potent entry inhibitors of SARS-CoV-2 virus infections. Eur J Med Chem..

[16] Huang Z.J., Zhang Y.H., Zhao L., Jing Y.W., Lai Y.S., Zhang L.Y., Guo Q., Yuan S., Zhang J., Peng S. (2010). Synthesis and anti-human hepatocellular carcinoma activity of new nitric oxide-releasing glycosyl derivatives of oleanolic acid. Org. Biomol. Chem..

[17] Meng L., Su Y., Yang F., Xiao S., Yin Z., Liu J., Zhong J., Zhou D., Yu F. (2019). Design, synthesis and biological evaluation of amino acids-oleanolic acid conjugates as influenza virus inhibitors. Bioorg. Med. Chem..

[18] Su Y., Meng L., Sun J., Li W., Shao L., Chen K., Zhou D., Yang F., Yu F. (2019). Design, synthesis of oleanolic acid-saccharide conjugates using click chemistry methodology and study of their anti-influenza activity. Europ. J. Med. Chem..

[19] Shao L., Yang F., Su Y., Li W., Zhang J., Xu H., Huang B., Sun M., Mu Y., Zhang Y. (2021). Design and Synthesis of Oleanolic Acid Trimers to Enhance Inhibition of Influenza Virus Entry. ACS Med. Chem. Lett..

[20] Özdemir Z., Bildziukevich U., Čapková M., Lovecká P., Rárová L., Šaman D., Zgarbova M., Lapuníkova B., Weber J., Kazakova O. (2021). Triterpenoid–PEG ribbons targeting selectivity in pharmacological effects. Biomedicines..

[21] Heller L., Knorrscheidt A., Flemming F., Wiemann J., Sommerwerk S., Pavel I.Z., Al-Harrasi A., Csuk R. (2016). Synthesis and proapoptotic activity of oleanolic acid derived amides. Bioorg. Chem..

[22] Spivak A.Y., Khalitova R.R., Nedopekina D.A., Gubaidullin R.R. (2020). Antimicrobial properties of amine- and guanidine-functionalized derivatives of betulinic, ursolic and oleanolic acids: Synthesis and structure-activity evaluation. Steroids.

[23] Vida N., Svobodová H., Rárová L., Drašar P., Šaman D., Cvačka J., Wimmer Z. (2012). Polyamine conjugates of stigmasterol. Steroids.

[24] Bildziukevich U., Malík M., Özdemir Z., Rárová L., Janovská L., Šlouf M., Šaman D., Šarek J., Nonappa N., Wimmer Z. (2020). Spermine amides of selected triterpenoid acids: Dynamic supramolecular system formation influences the cytotoxicity of the drugs. J. Mater. Chem. B..

[25] Bildziukevich U., Kaletová E., Šaman D., Sievänen E., Kolehmainen E.T., Šlouf M., Wimmer Z. (2017). Spectral and microscopic study of self-assembly of novel cationic spermine amides of betulinic acid. Steroids.

[26] Kazakova O.B., Brunel J.M., Khusnutdinova E.F., Negrel S., Giniyatullina G.V., Lopatina T.V., Petrova A.V. (2019). A-Ring-modified triterpenoids and their spermidine–aldimines with strong antibacterial activity. Molblank.

[27] Khusnutdinova E.F., Sinou V., Babkov D.A., Kazakova O.B., Brunel J.M. (2022). Development of New Antimicrobial Oleanonic Acid Polyamine Conjugates. Antibiotics.

[28] Kazakova O.B., Giniuatullina G.V., Babkov D.V., Wimmer Z. (2022). From Marine Metabolites to the Drugs of the Future: Squalamine, Trodusquemine, Their Steroid and Triterpene Analogues. Int. J. Mol. Sci..

[29] Özdemir Z., Šaman D., Bednárová L., Pazderková M., Janovská L., Nonappa N., Wimmer Z. (2022). Aging-Induced Structural Transition of Nanoscale Oleanolic Acid Amphiphiles and Selectivity against Gram-Positive Bacteria. ACS Appl. Nano Mater..

[30] Hao Y., Hua D., Miao T., Wang S., Jin X., Gu W. (2016). Synthesis, crystal structure and antitumor activity of a new indolequinone derivative of ursolic acid. Chin. J. Struct. Chem..

[31] Gupta N., Rath S.K., Singh J., Qayum A., Singh S., Sangwan P.L. (2017). Synthesis of novel benzylidene analogues of betulinic acid as potent cytotoxic agents. Eur. J. Med. Chem..

[32] Khusnutdinova E., Galimova Z., Lobov A., Baikova I., Kazakova O., Thu H.N.T., Tuyen N.V., Gatilov Y., Csuk R., Serbian S. (2021). Synthesis of messagenin and platanic acid chalcone derivatives and their biological potential. Nat. Prod. Res..

[33] Khusnutdinova E.F., Petrova A.V., Kukovinets O.S., Kazakova O.B. (2018). Synthesis and cytotoxicity of 28-*N*-propargylaminoalkylated 2,3-indolotriterpenic acids. Nat. Prod. Commun..

[34] Khusnutdinova E., Petrova A., Zileeva Z., Kuzmina U., Zainullina L., Vakhitova Y., Babkov D., Kazakova O. (2021). Novel A-ring chalcone derivatives of oleanolic and ursolic amides with anti-proliferative effect mediated through ROS-triggered apoptosis. Int. J. Mol. Sci..

[35] Kazakova O., Rubanik L., Lobov A., Poleshchuk N., Baikova I., Kapustina Y., Petrova A., Korzun T., Lopatina T., Fedorova A. (2021). Synthesis of erythrodiol C-ring derivatives and their activity against *Chlamydia trachomatis*. Steroids.

[36] Wang W., Lei L., Liu Z., Wang H., Meng Q. (2019). Design, synthesis, and biological evaluation of novel nitrogen heterocycle-containing ursolic acid analogs as antitumor agents. Molecules.

[37] Kazakova O.B., Medvedeva N.I., Samoilova I.A., Baikova I.P., Tolstikov G.A., Kataev V.E., Mironov V.F. (2011). Conjugates of several lupane, oleanane, and ursane triterpenoids with the antituberculosis drug isoniazid and pyridinecarboxaldehydes. Chem. Nat. Compd..

[38] Wrzeciono U., Turowska W., Gorczynska L. (1973). Nitrogen derivatives of triterpenes. VII. Products of the reduction of oleanonic acid oxime and its methyl ester. Roczniki Chem..

[39] Ghose A.K., Viswanadhan V.N., Wendoloski J.J.J. (1999). A knowledge-based approach in designing combinatorial or medicinal chemistry libraries for drug discovery. 1. A qualitative and quantitative characterization of known drug databases. Combinat. Chem..

[40] Song G., Shen X., Li S., Li Y., Liu Y., Zheng Y., Lin R., Fan J., Ye H., Liu S. (2015). Structure-activity relationships of 3-*O*-beta-chacotriosyl ursolic acid derivatives as novel H5N1 entry inhibitors. Eur. J. Med. Chem..

[41] Boreko E.I., Pavlova N.I., Savinova O.V., Nikolaeva S.N., Flekhter O.B., Phyzhova N.S. (2002). Inhibition of virus reproduction and proteinase activity by lupane and some other terpenes. J. Biomed. Sci..

[42] Flekhter O.B., Ashavina O.Y., Smirnova I.E., Baltina L.A., Galin F.Z., Kabal’nova N.N., Tolstikov G.A. (2004). Selective oxidation of triterpene alcohols by sodium hypochlorite. Chem. Nat. Comp..

[43] Smirnova I.E., Kazakova O.B. (2018). Structure—Anti-influenza type A activity relationship among a series of nitrogen lupane triterpenoids. Nat. Prod. Comm..

[44] Tolmacheva I.A., Igosheva E.V., Savinova O.V., Boreko E.I., Grishko V.V. (2014). Synthesis and antiviral activity of C-3(C-28)-substituted 2,3-*seco*-triterpenoids. Chem. Nat. Compd..

[45] Gupta S., Kalani K., Saxena M., Srivastava S.K., Agrawal S.K., Suri N., Saxena A.K. (2010). Cytotoxic Evaluation of Semisynthetic Ester and Amide Derivatives of Oleanolic Acid. Nat. Prod. Comm..

[46] Hodon J.L., Pokorny J., Kazakova A., Urban M. (2019). Design and synthesis of pentacyclic triterpene conjugates and their use in medicinal research. Eur. J. Med.Chem..

[47] Smirnova I., Petrova A., Lobov A., Minnibaeva E., Tran T.P.T., Tran V.L., Khine M., Esulakova I., Slita A., Zarubaev V. (2022). Azepanodipterocarpol is potential candidate for inhibits influenza H1N1 type among other lupane, oleanane, and dammarane A-ring amino-triterpenoids. J. Antibiot..

[48] Pęcak P., Orzechowska B., Chrobak E., Boryczka S. (2021). Novel betulin dicarboxylic acid ester derivatives as potent antiviral agents: Design, synthesis, biological evaluation, structure-activity relationship and *in-silico* study. Eur. J. Med. Chem..

[49] Alvarenga T.A., Bêdo T.R.O., Braguine C.G., Gonçalves U.O., Magalhães L.G., Rodrigues V., Gimenez V.M.M., Groppo M., Silva M.L.A., Cunha W.R. (2012). Evaluation of *Cuspidaria pulchra* and its isolated compounds against *Schistosoma mansoni* Adult Worms. Int. J. Biotechn. Wellness Ind..

[50] Crance J.M., Scaramozzino N., Jouan A., Garin D. (2003). Interferon, Ribavirin, 6-Azauridine and Glycyrrhizin: Antiviral Compounds. Active against Pathogenic Flaviviruses. Antivir. Res..

[51] Baltina L.A., Tasi Y.-T., Huang S.-H., Lai H.-C., Baltina L.A., Petrova S.F., Yunusov M.S., Lin C.-W. (2019). Glycyrrhizic acid derivatives as Dengue virus inhibitors. Bioorg. Med. Chem. Lett..

[52] Hour M.-J., Chen Y., Lin C.-S., Baltina L.A., Kan J.-Y., Tsai Y.-T., Kiu Y.-T., Lai H.-C., Baltina L.A., Petrova S.F. (2022). Glycyrrhizic Acid Derivatives Bearing Amino Acid Residues in the Carbohydrate Part as Dengue Virus E Protein Inhibitors: Synthesis and Antiviral Activity. Int. J. Mol. Sci..

[53] https://www.the-easia.org/jrp/pdf/cfp09/easia_9th_selected_projects_health.pdf.

[54] Vistica D.T., Skehan P., Scudiero D., Monks A., Pittman A., Boyd M.R. (1991). Tetrazolium-based assays for cellular viability: A critical examination of selected parameters affecting formazan production. Cancer Res..

[55] Scudiero D.A., Shoemaker R.H., Paull K.D., Monks A., Tierney S., Nofziger T.H., Currens M.J., Seniff D., Boyd M.R. (1988). Evaluation of a soluble tetrazolium/formazan assay for cellgrowth and drug sensitivity in culture using human and other tumor cell lines. Cancer Res..

