# Supplementary material for: Synthesis, Anti-Influenza H1N1 and Anti-Dengue Activity of A-Ring Modified Oleanonic Acid Polyamine Derivatives
Source: Molecules. 2022 Dec 2;27(23):8499. doi: 10.3390/molecules27238499 (PMC9738632; doi:10.3390/molecules27238499)

Synthesis, anti-influenza H1N1 and anti-Dengue activity of A-ring modified oleanonic acid polyamine derivatives

Irina Smirnova[1], Anastasiya Petrova[1], Gul'nara Giniyatullina[1], Anna Smirnova[1], Alexandrina Volobueva[2], Julia Pavlyukova[3], Vladimir Zarubaev[4], Tran Van Loc[4], Thao Tran Thi Phoung[4], Vu Thi Bich Hau[5], Nguyen Thi Thu Thuy[5], Myint Myint Khine[6] and Oxana Kazakova[1*]

[1] Ufa Institute of Chemistry of the Ufa Federal Research Centre of the Russian Academy of Sciences, 71, pr. Oktyabrya, 450054 Ufa, Russian Federation; si8081@yandex.ru (I.S.); ana.orgchem@gmail.com (A.P.); gulnaravlg@gmail.com (G.G.); bazunova03@yandex.ru (A.S.); obf@anrb.ru (O.K.)
[2] Department of virology, St. Petersburg Pasteur Institute of Epidemiology and Microbiology, Experimental virology laboratory, St. Petersburg, 14 Mira St., 197001, Russian Federation; sasha-khrupina@mail.ru (A.V.); zarubaev@gmail.com (V.Z.)
[3] St.-Petersburg State Institute of Technology, 26 Moskovsky Av, St. Petersburg 190013, Russia; julia_pavljukova@mail.ru (J.P.)
[4] Institute of Chemistry – Vietnamese Academy of Science and Technology 18- Hoang Quoc Viet street, Cau Giay district, Hanoi, 1000, Vietnam; tvloc61@gmail.com (T.V.L.); ntuelam2010@gmail.com (T.T.T.P.)
[5] National Institute of Hygiene and Epidemiology, 1 Yersin street, Hai Ba Trung, Hanoi 10000, Vietnam; hauvtb@gmail.com (V.T.B.H.); ticun_2002@yahoo.com (N.T.T.T.)
[6] Department of Chemistry, University of Yangon, University Avenue Road, Kamayut Township 11041, Yangon, Myanmar; mmkhine2005@googlemail.com (M.M.K.)
* Correspondence: obf@anrb.ru (O.K.) Tel: +7(347)2-35-6066

Abstract: A series of sixteen A-ring modified (2,3-indolo-, 2-benzylidene) oleanonic acid derivatives holding some cyclic amines, linear polyamines and benzylaminocarboxamides at C28 has been synthesized and screened for antiviral activity against influenza A/PuertoRico/8/34 (H1N1) and Dengue virus serotypes of DENV-1, -2, -3, -4. It was found that 28- homopiperazine **2** and 3-*N*-phthalyl **22** amides of oleanonic acid demonstrated high potency with selectivity index SI 27 (IC_{50} 21 μM) and 42 (IC_{50} 12 μM). Oleanonic acid aminoethylpiperazine amide **6** and C-azepano-erythrodiol **23** appeared to be the most effective compounds against DENV-1 (IC_{50}'s 67 and 107 μM) and -2 (IC_{50}'s 86 and 68 μM correspondingly) serotypes.

Keywords: triterpenoids; oleanolic acid; polyamine; Ugi derivatives; indole; benzylidene; antiviral activity; influenza A (H1N1); Dengue virus

Figure S1. ^{1}H and $^{13}C\{^{1}H\}$ NMR spectra of compound **2**

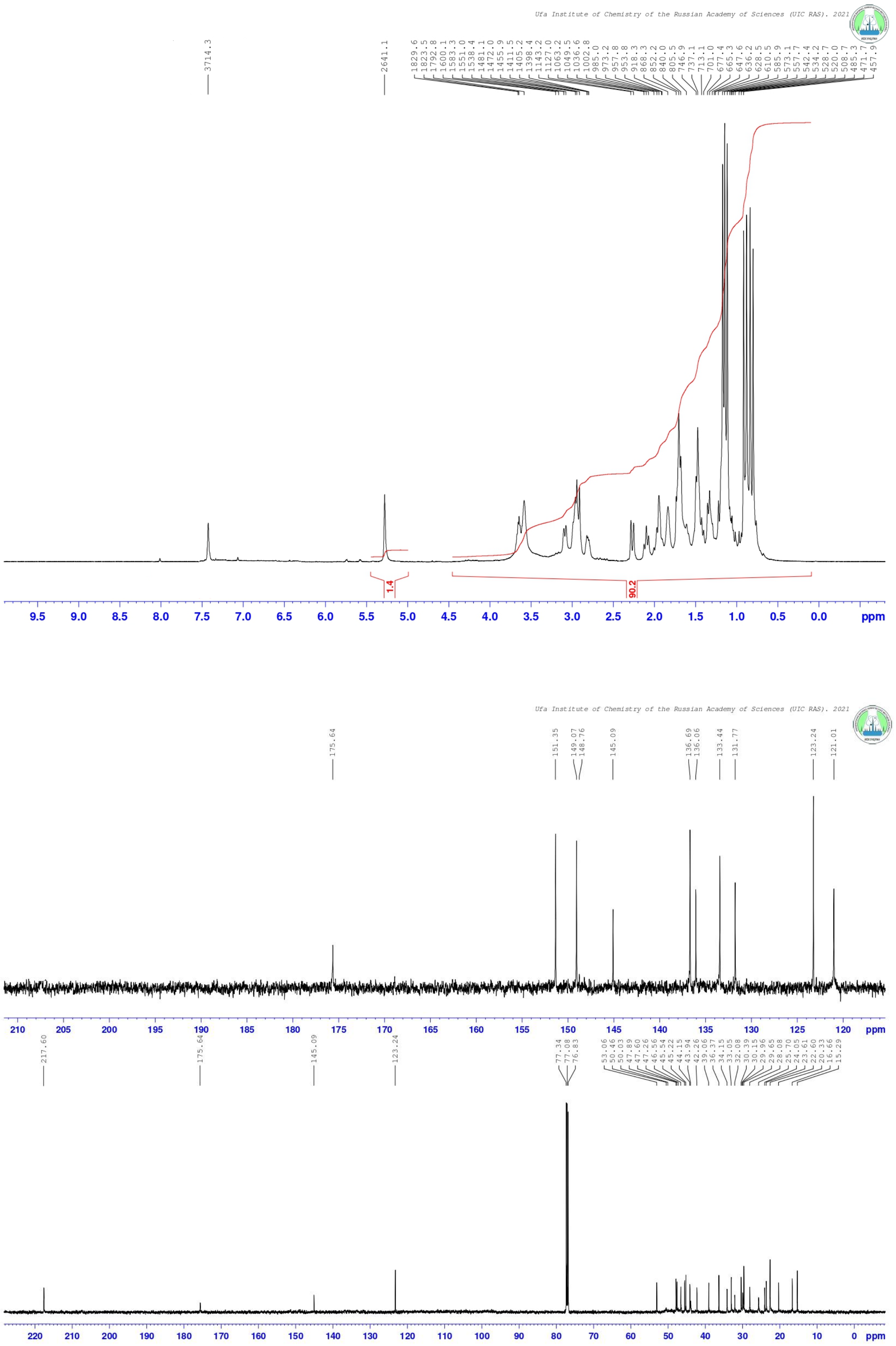

Figure S2. MS-APSI spectra of compound **2**

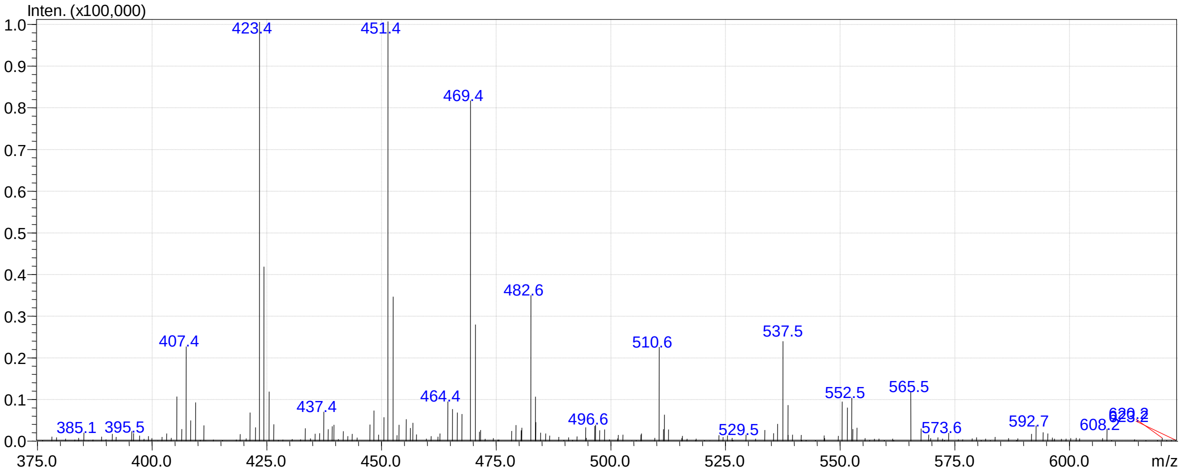

Figure S3. ^{1}H and $^{13}C\{^{1}H\}$ (DEPT-135 edited) NMR spectra of compound **3**

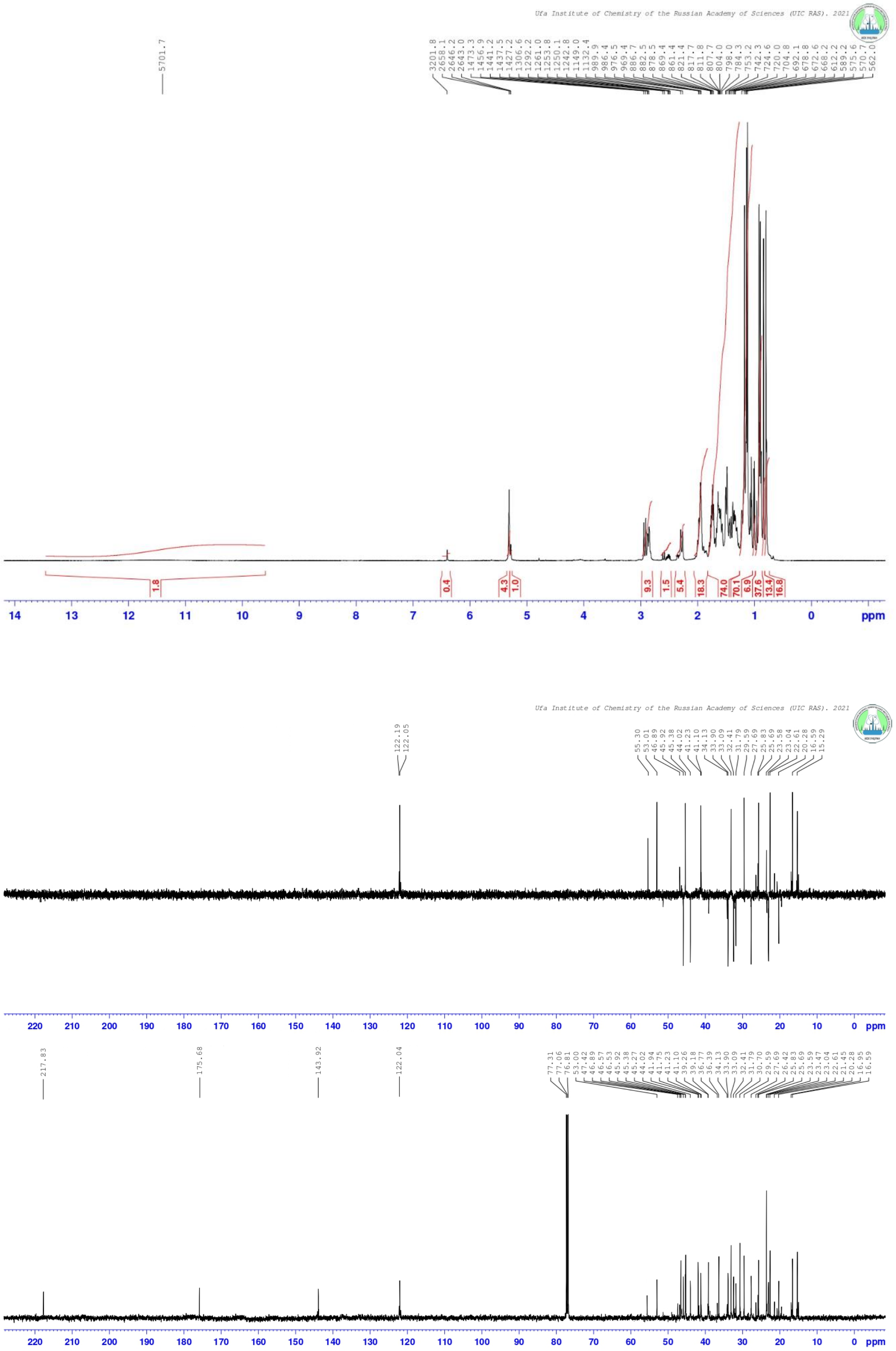

Figure S4. MS-APSI spectra of compound **3**

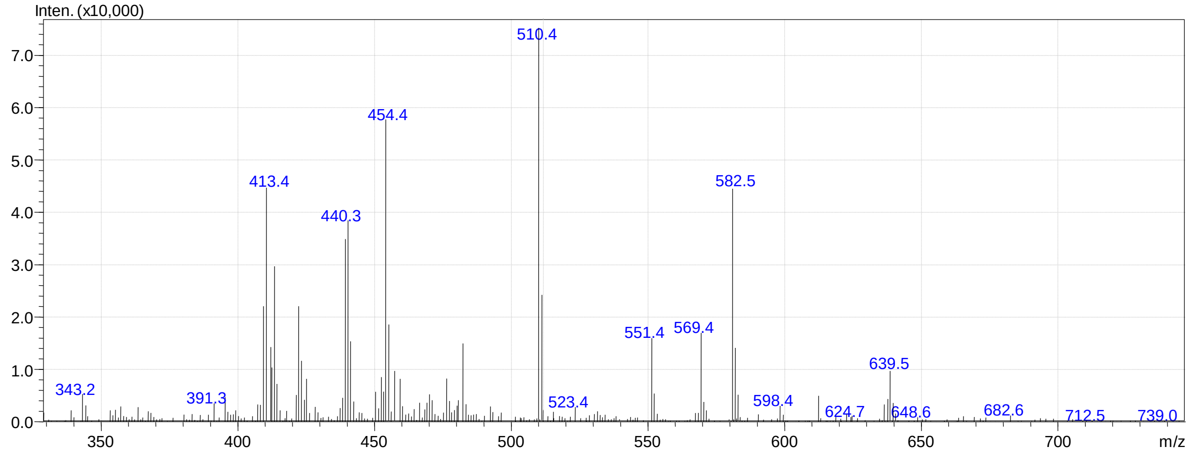

Figure S5. ^{1}H and $^{13}C\{^{1}H\}$ NMR spectra of compound **4**

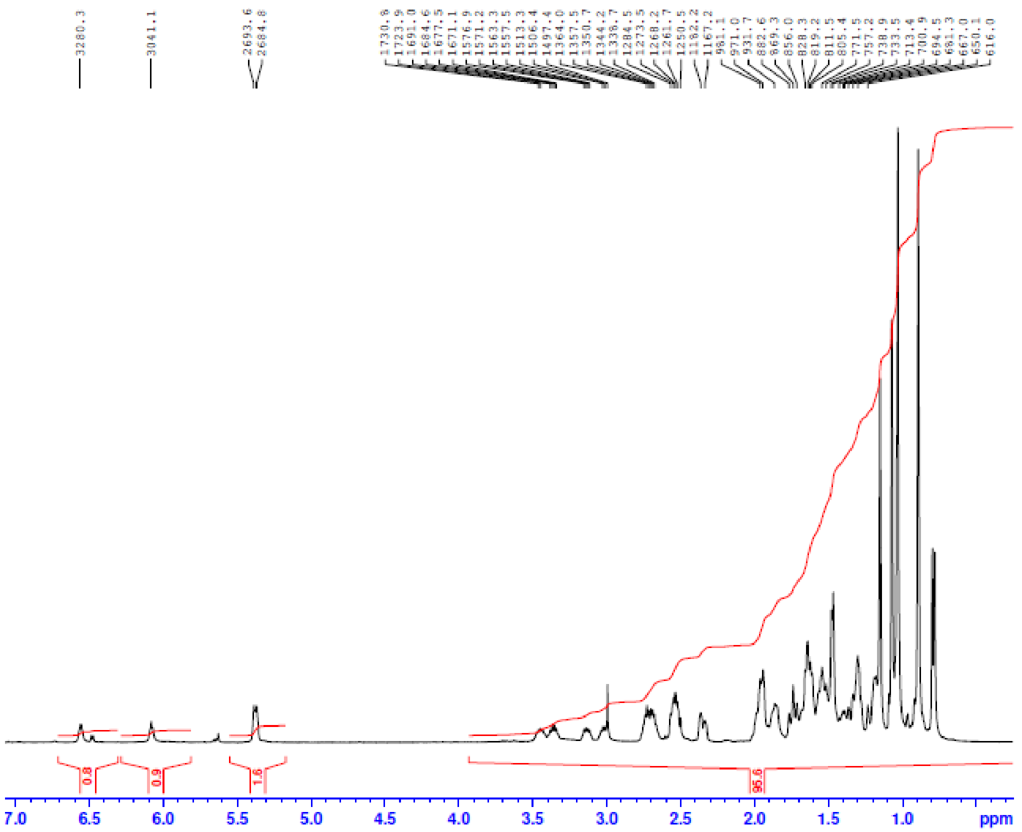

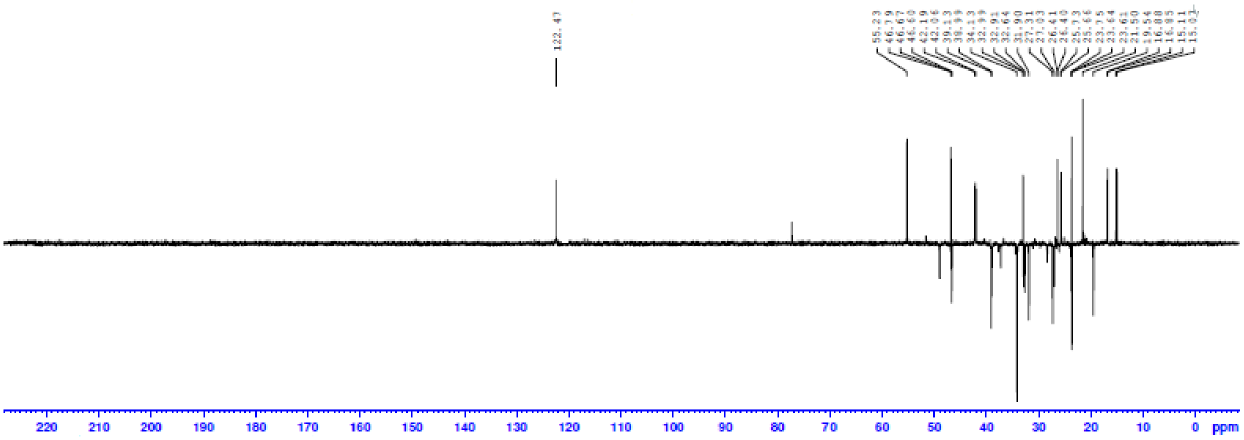

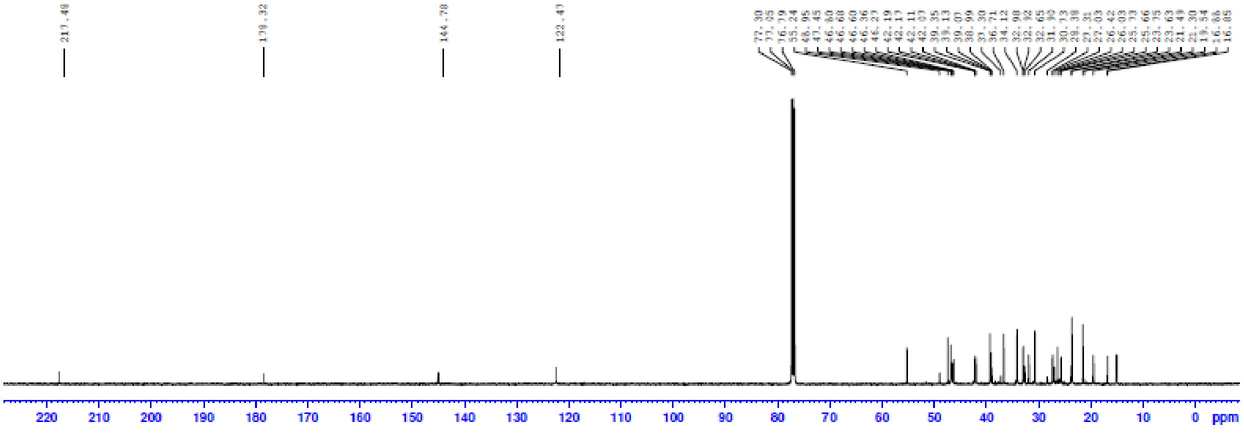

Figure S6. MS-APSI spectra of compound **4**

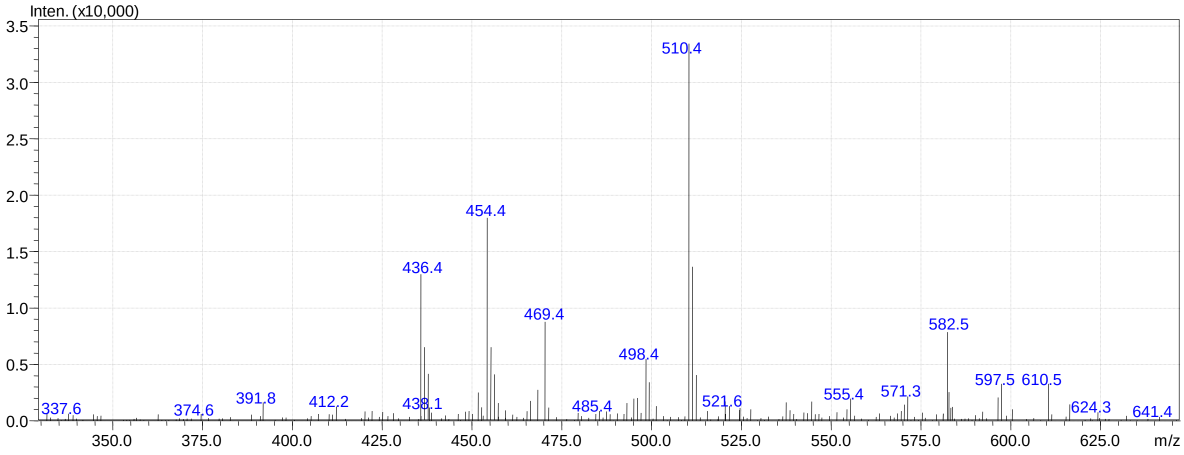

Figure S7. ^{1}H and ^{13}C{^{1}H} (DEPT-135 edited) NMR spectra of compound **6**

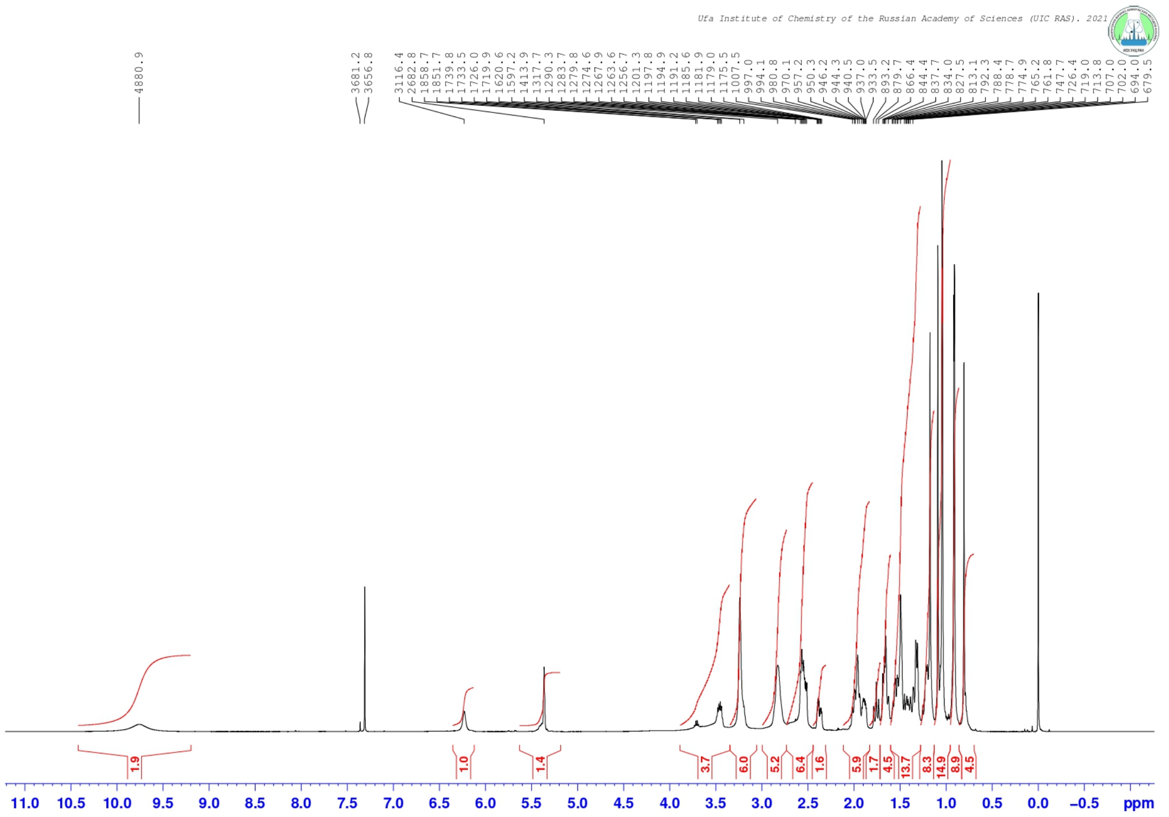

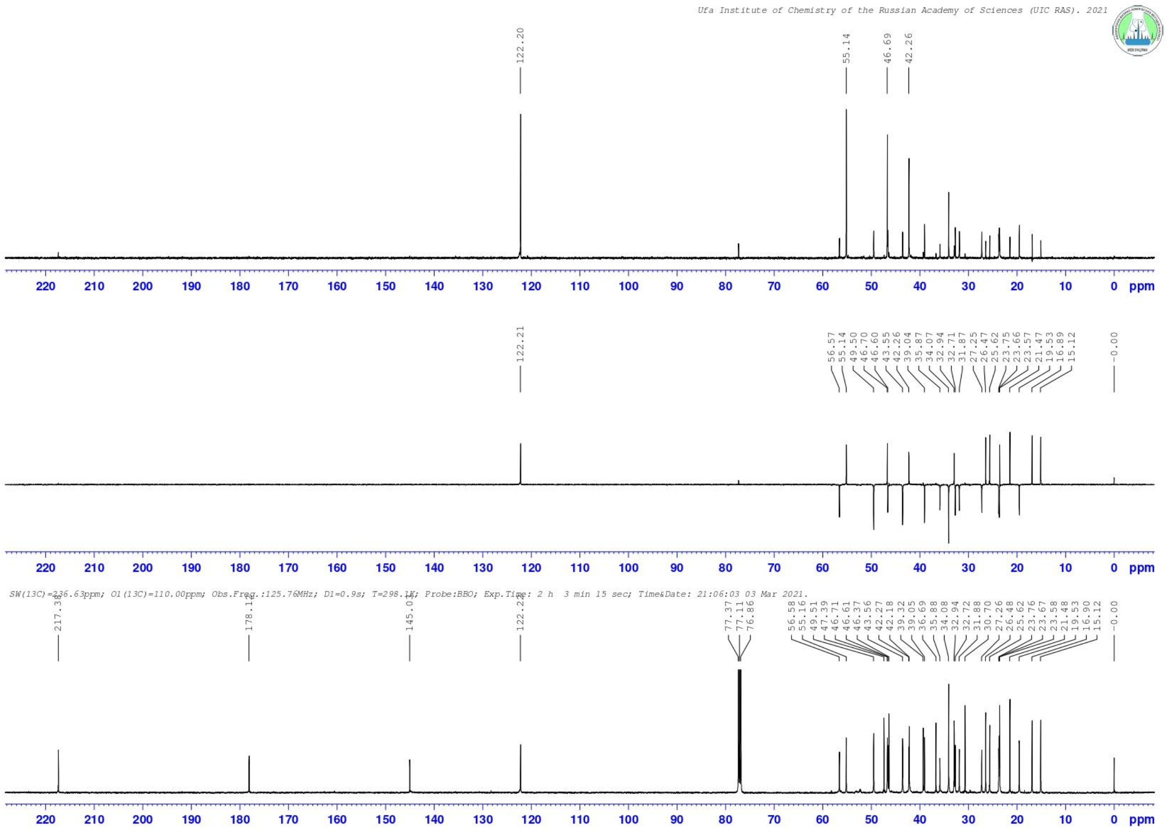

Figure S8. MS-APSI spectra of compound **6**

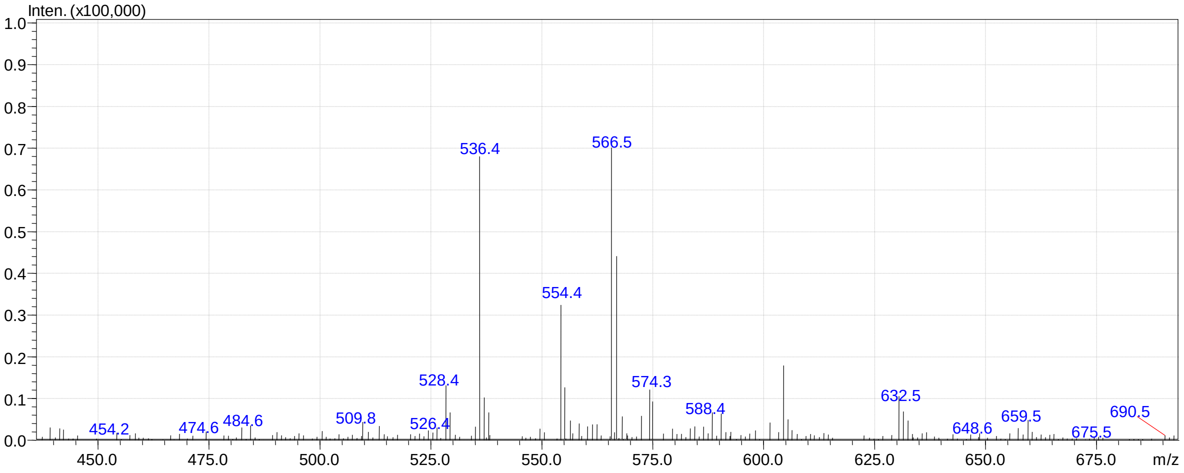

Figure S9. ^{1}H and $^{13}C\{^{1}H\}$ (DEPT-135 edited) NMR spectra of compound **7**

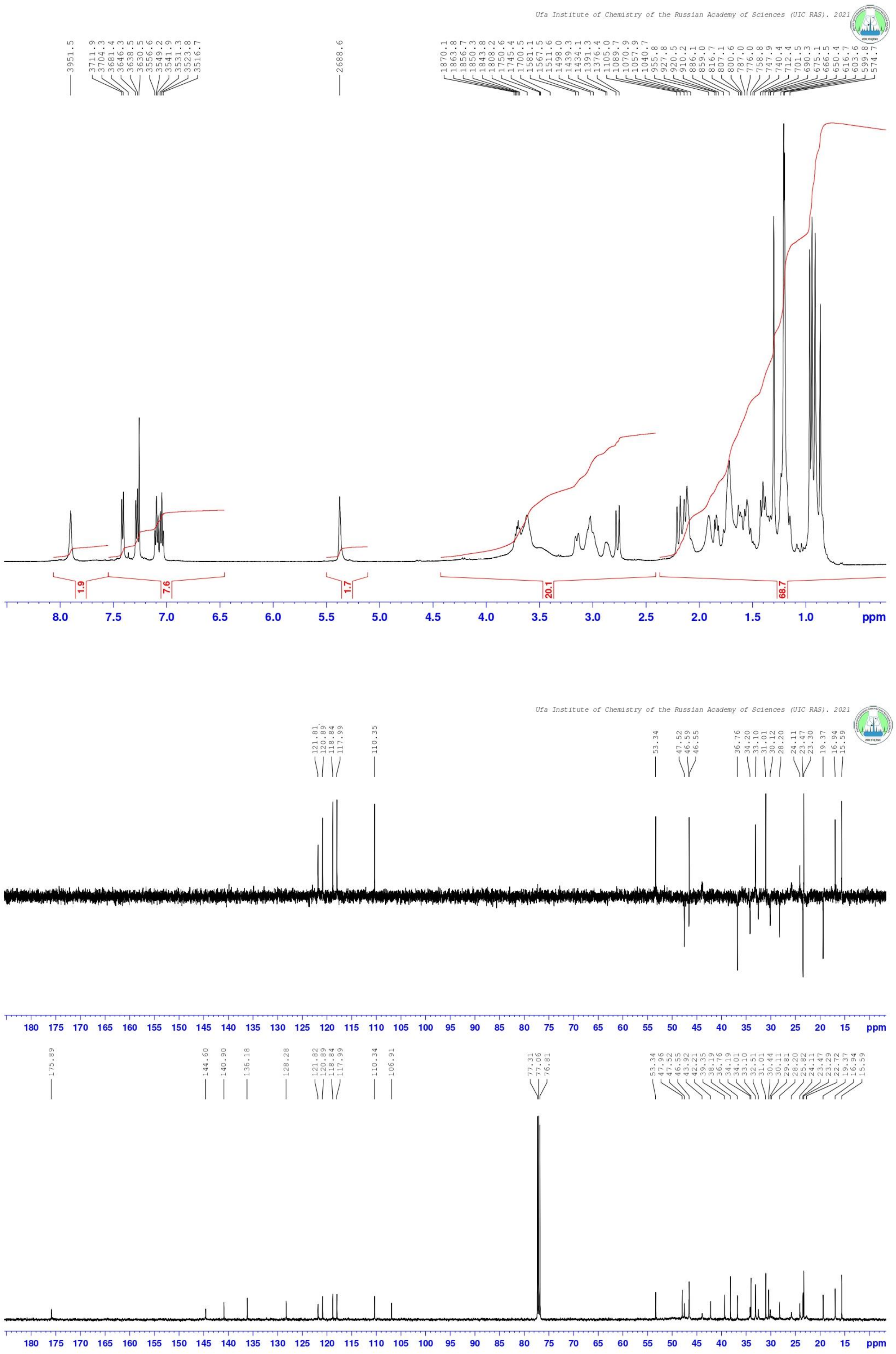

Figure S10. MS-APSI spectra of compound **7**

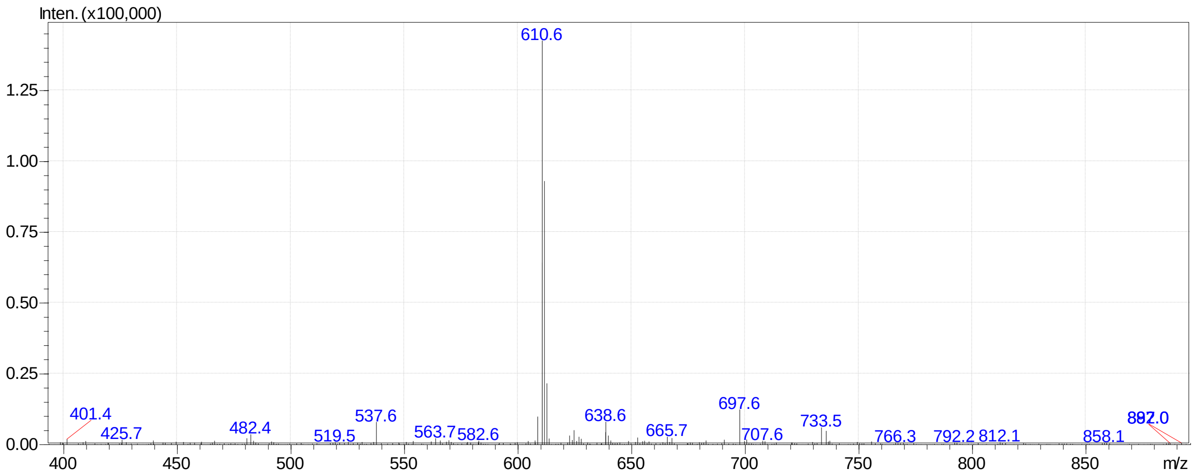

Figure S11. ^{1}H and $^{13}C\{^{1}H\}$ NMR spectra of compound **8**

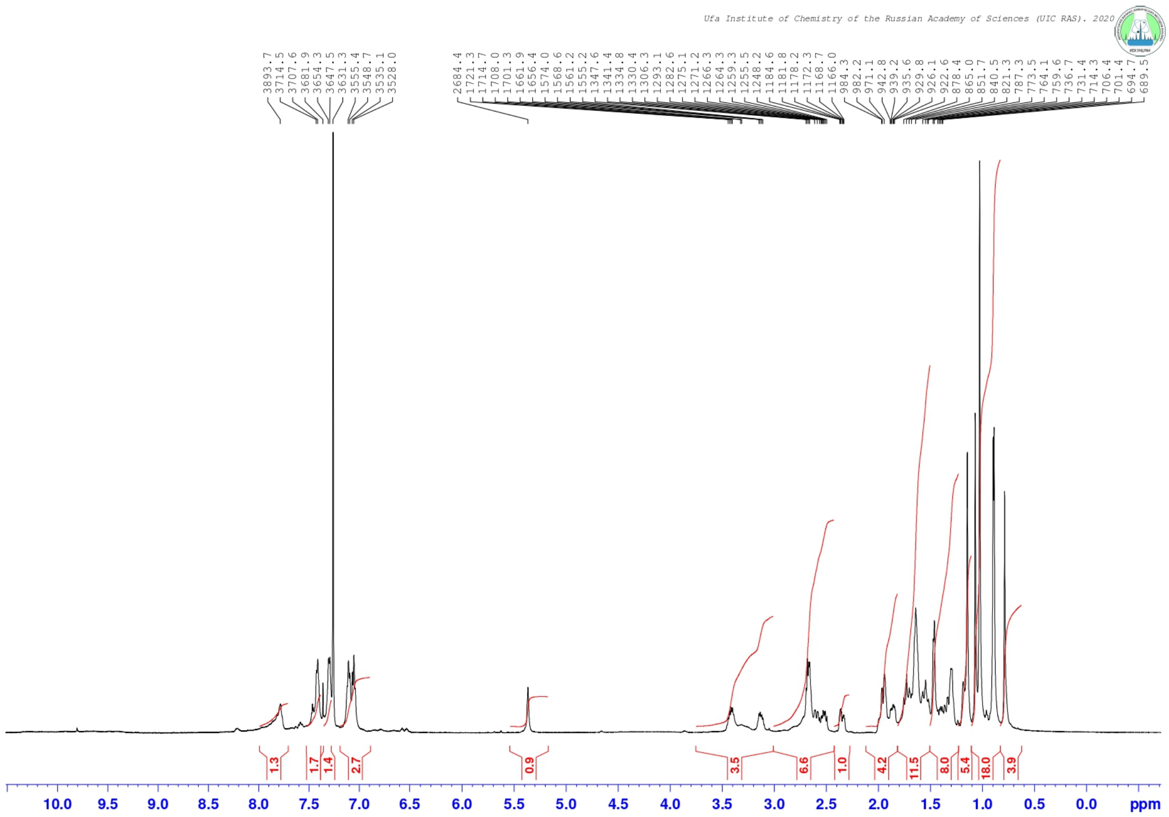

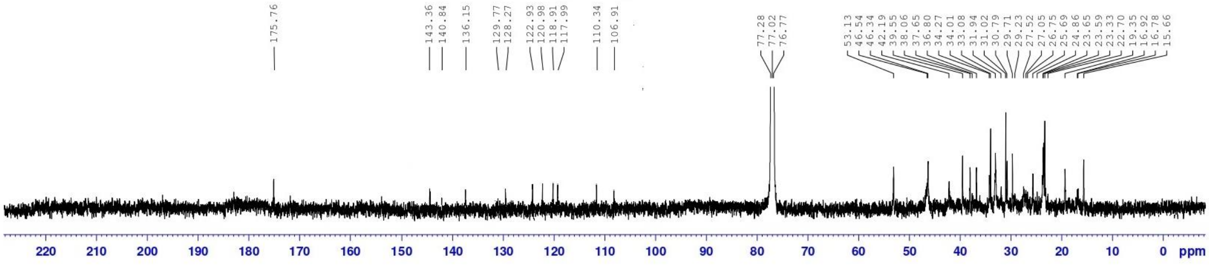

Figure S12. MS-APSI spectra of compound **8**

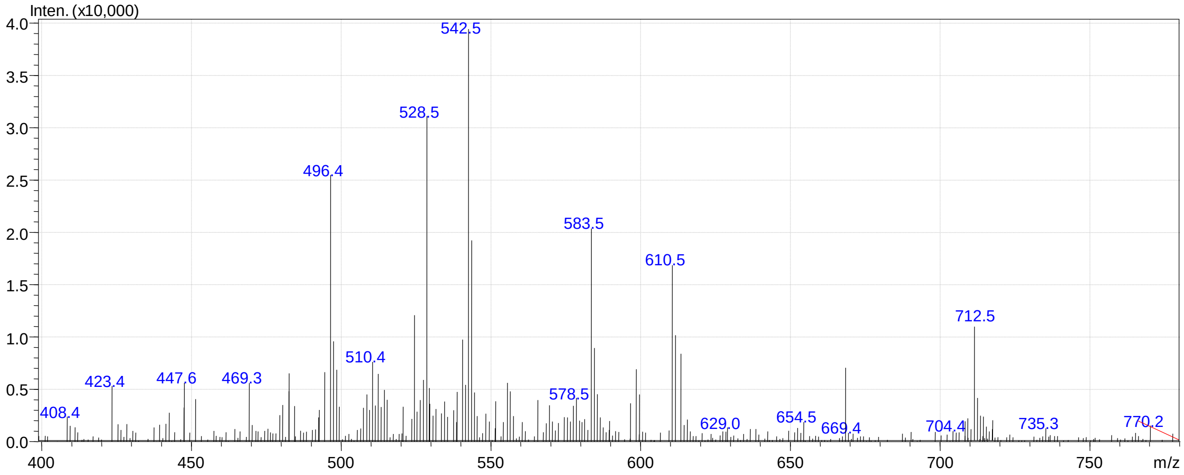

Figure S13. ^{1}H and ^{13}C{^{1}H} (DEPT-135 edited) NMR spectra of compound **9**

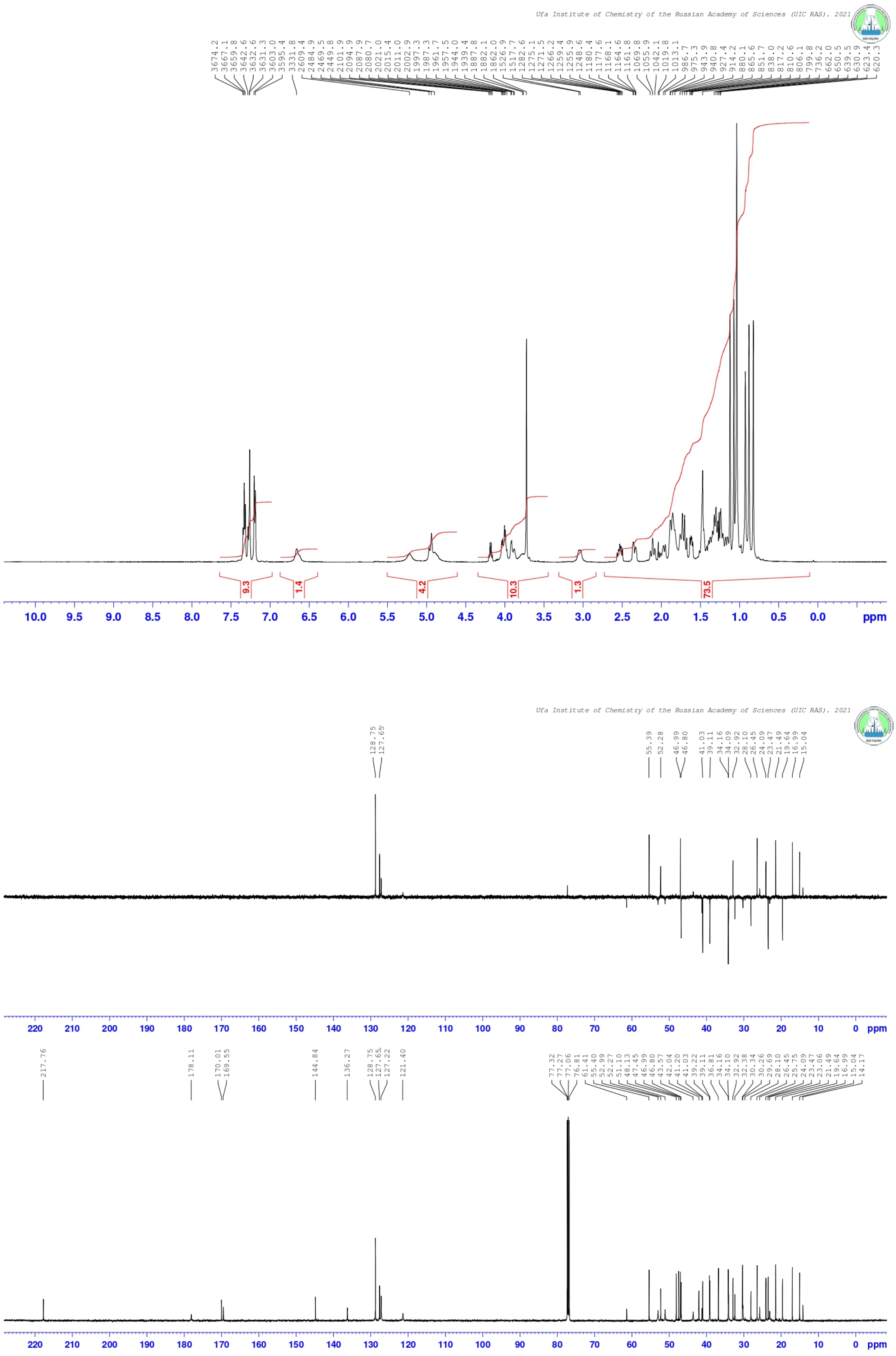

Figure S14. MS-APSI spectra of compound **9**

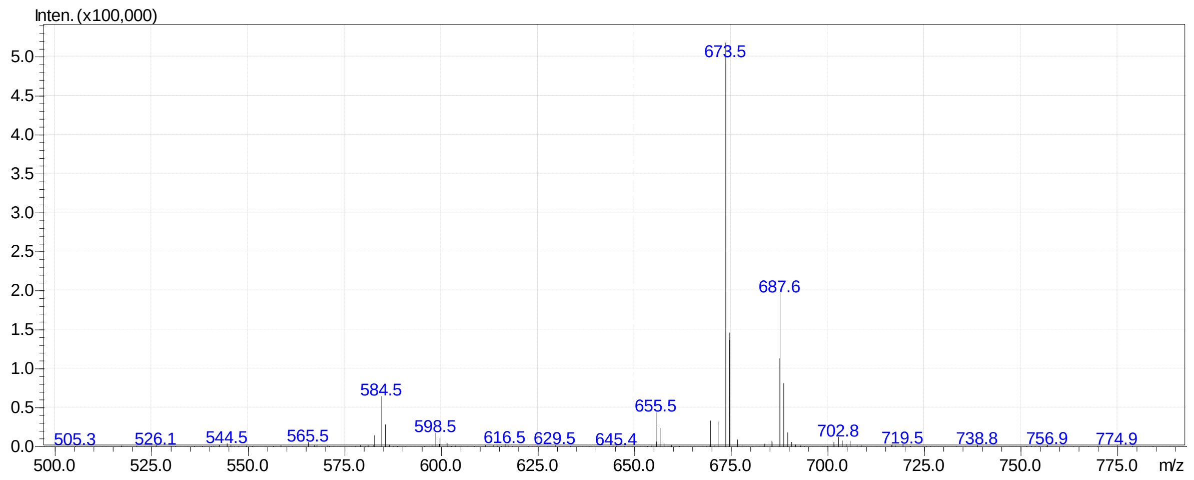

Figure S15. ^{1}H and $^{13}C\{^{1}H\}$ (DEPT-135 edited) NMR spectra of compound **10**

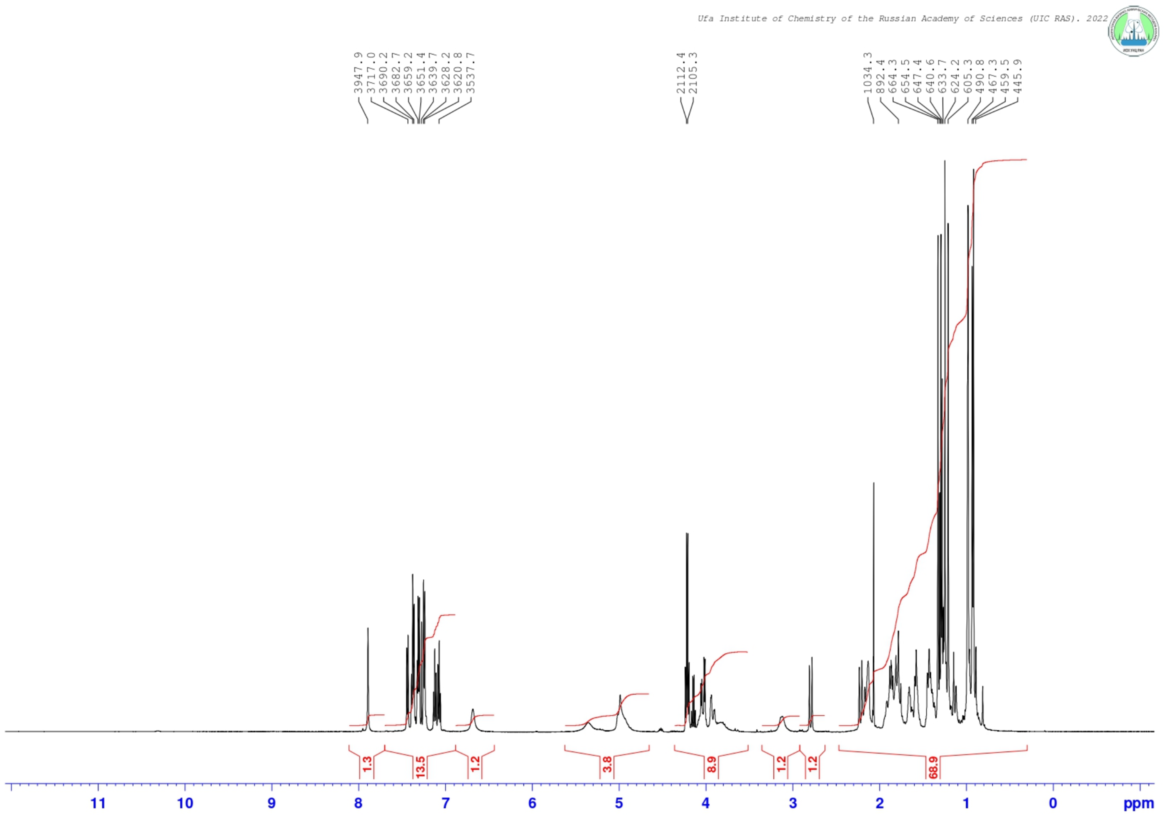

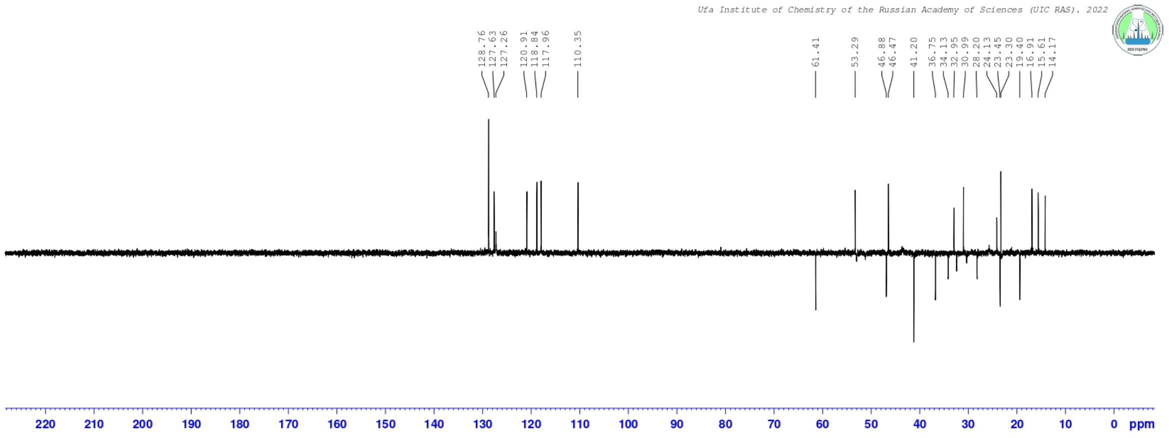

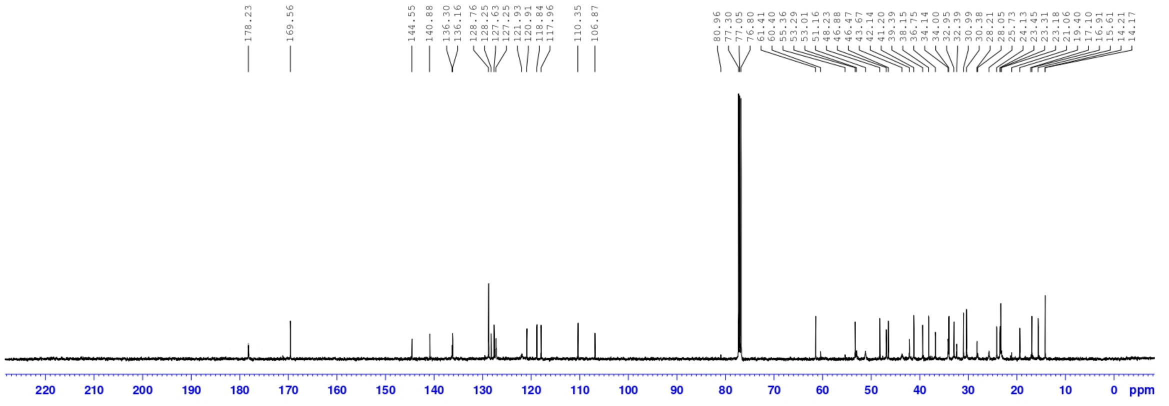

Figure S16. MS-APSI spectra of compound **10**

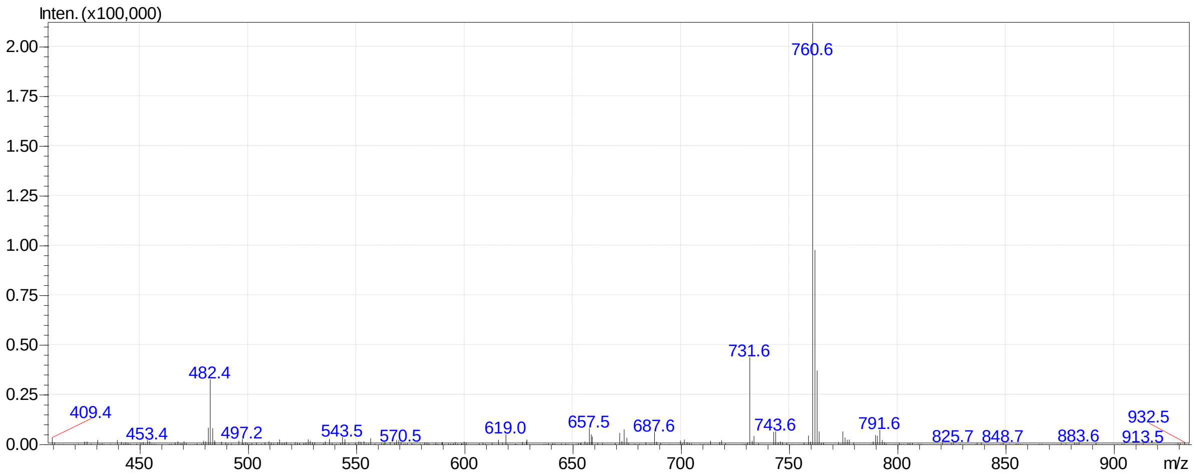

Figure S17. ^{1}H and $^{13}C\{^{1}H\}$ NMR spectra of compound **11**

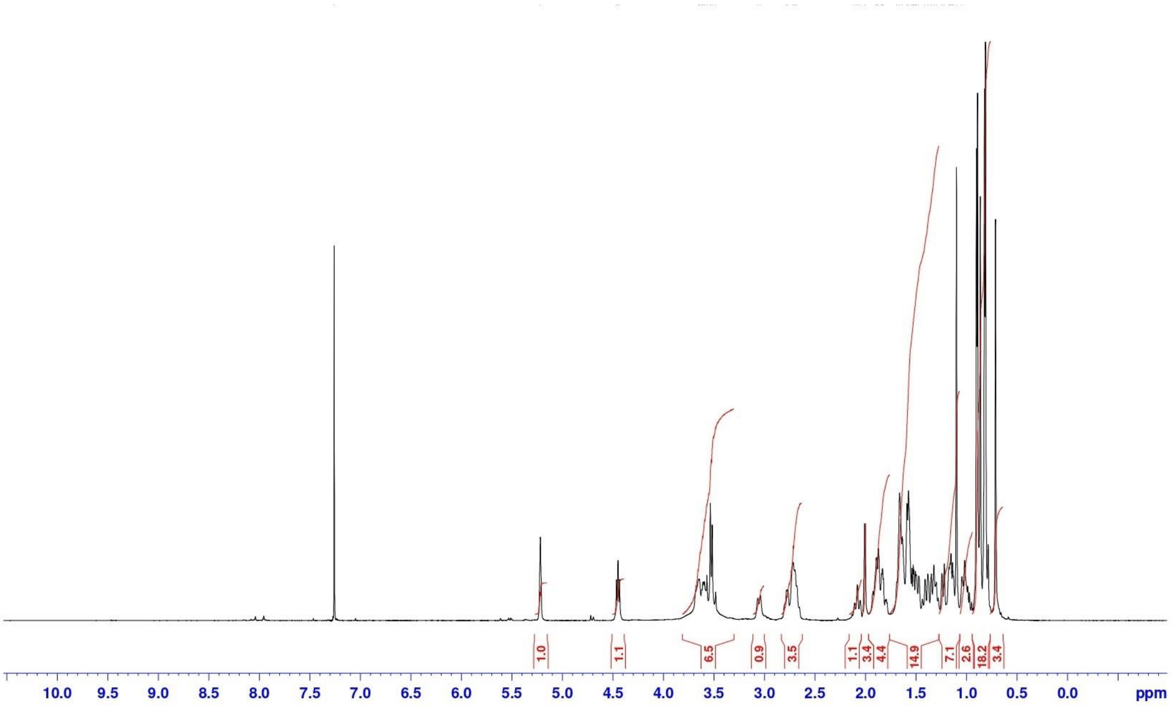

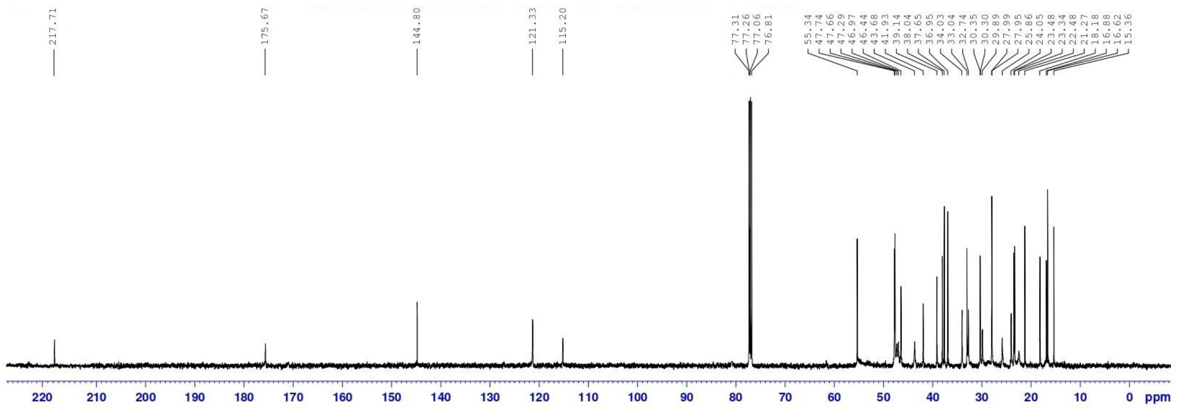

Figure S18. MS-APSI spectra of compound **11**

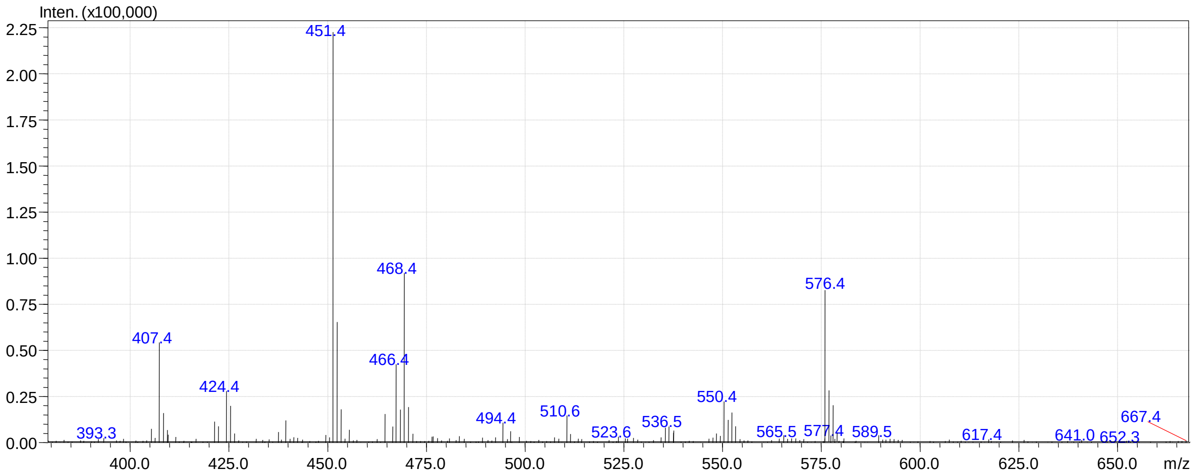

Figure S19. ^{1}H and ^{13}C{^{1}H} NMR spectra of compound **12**

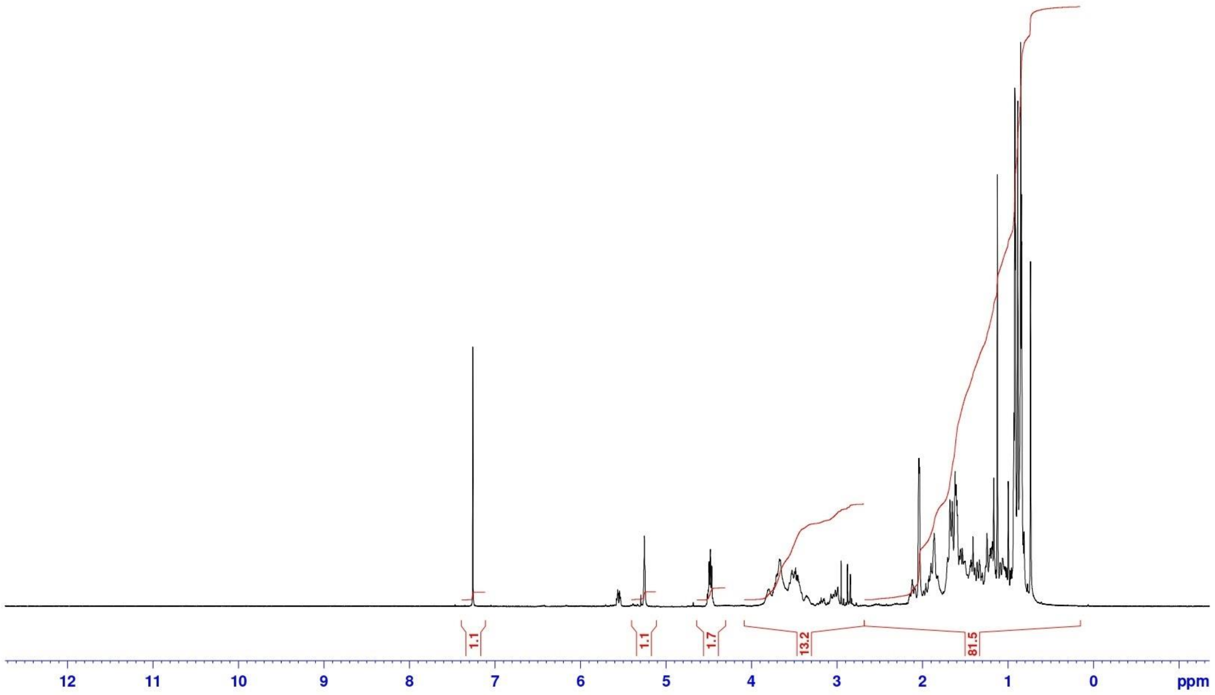

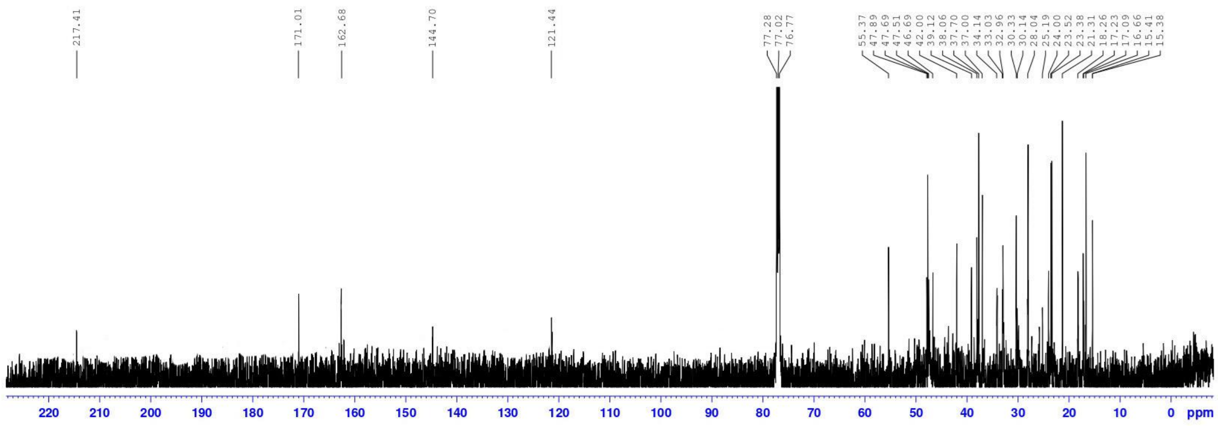

Figure S20. MS-APSI spectra of compound **12**

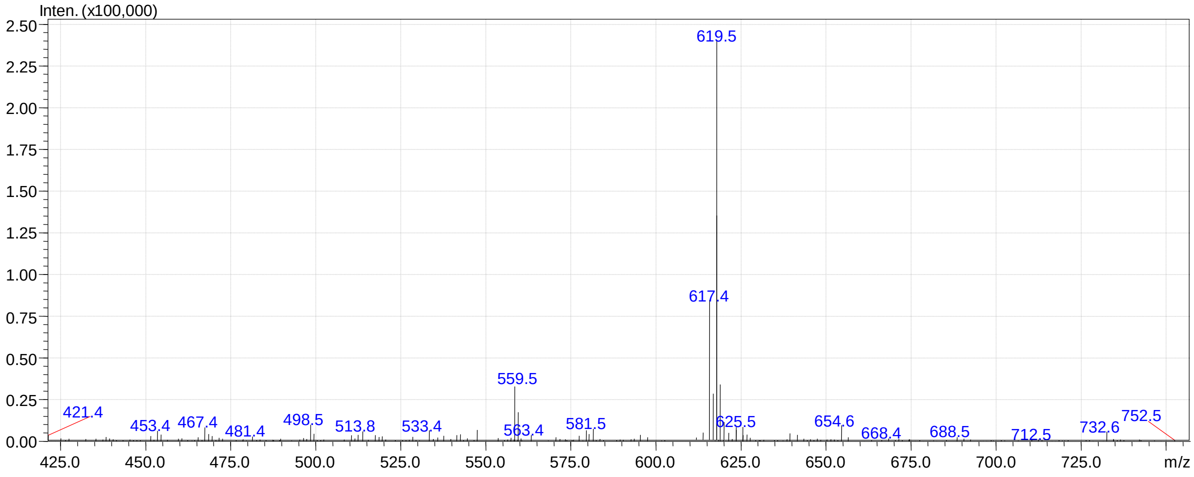

Figure S21. ^{1}H and $^{13}C\{^{1}H\}$ NMR spectra of compound **15**

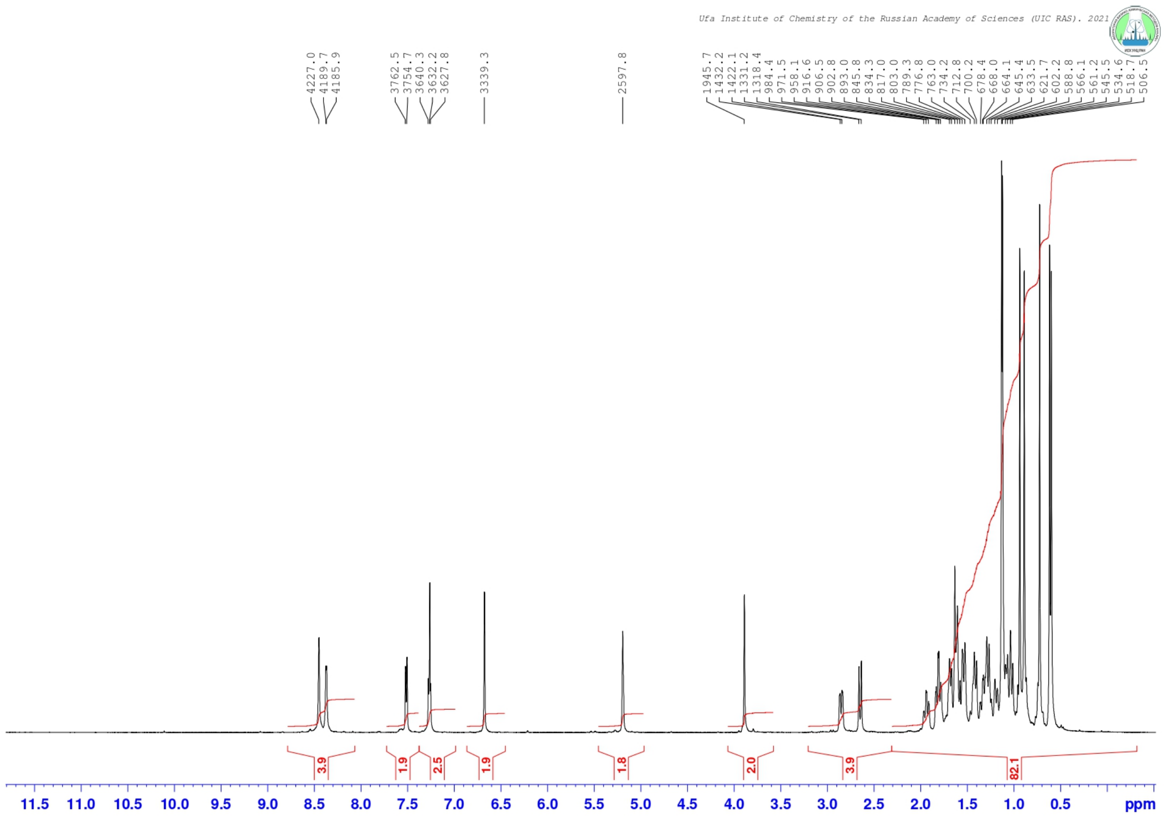

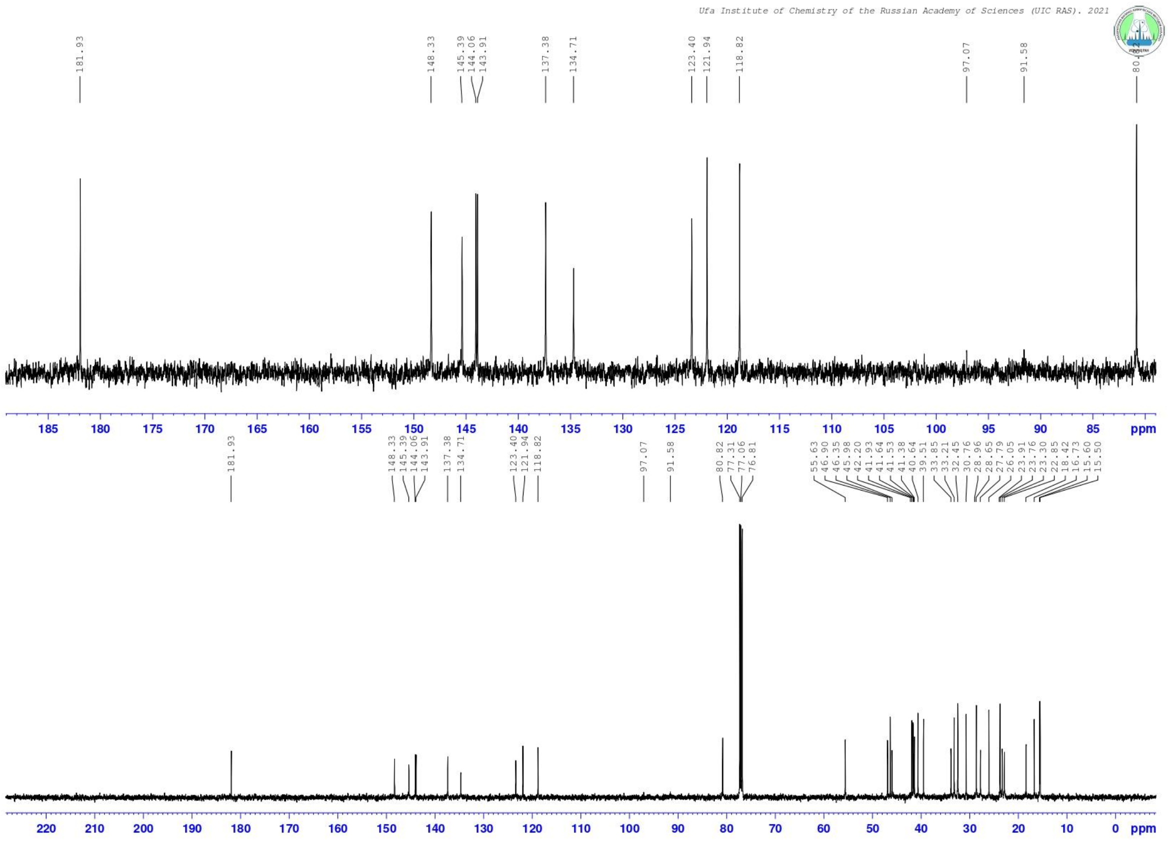

Figure S22. MS-APSI spectra of compound **15**

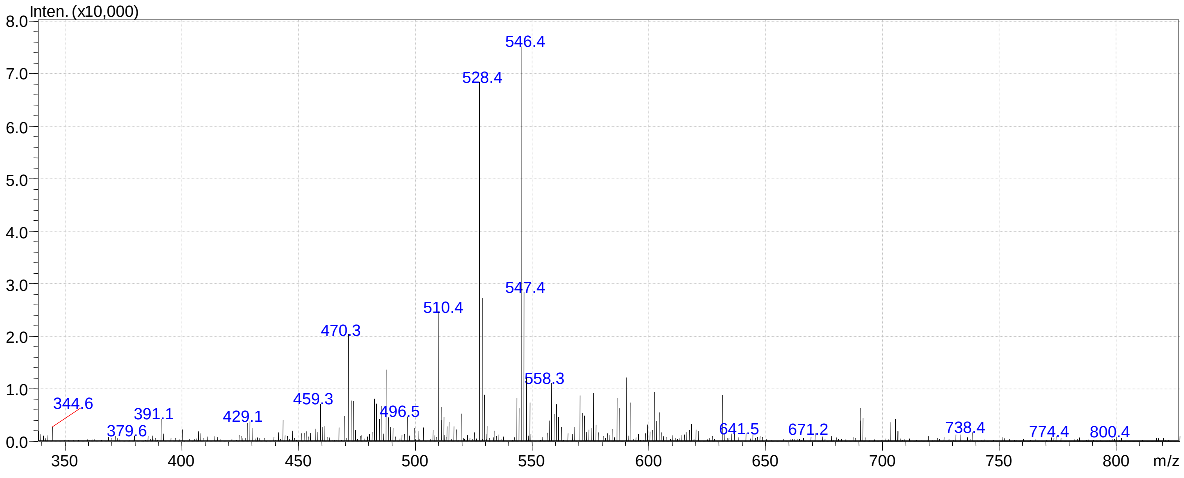

Figure S23. ^{1}H and $^{13}C\{^{1}H\}$ (DEPT-135 edited) NMR spectra of compound **16**

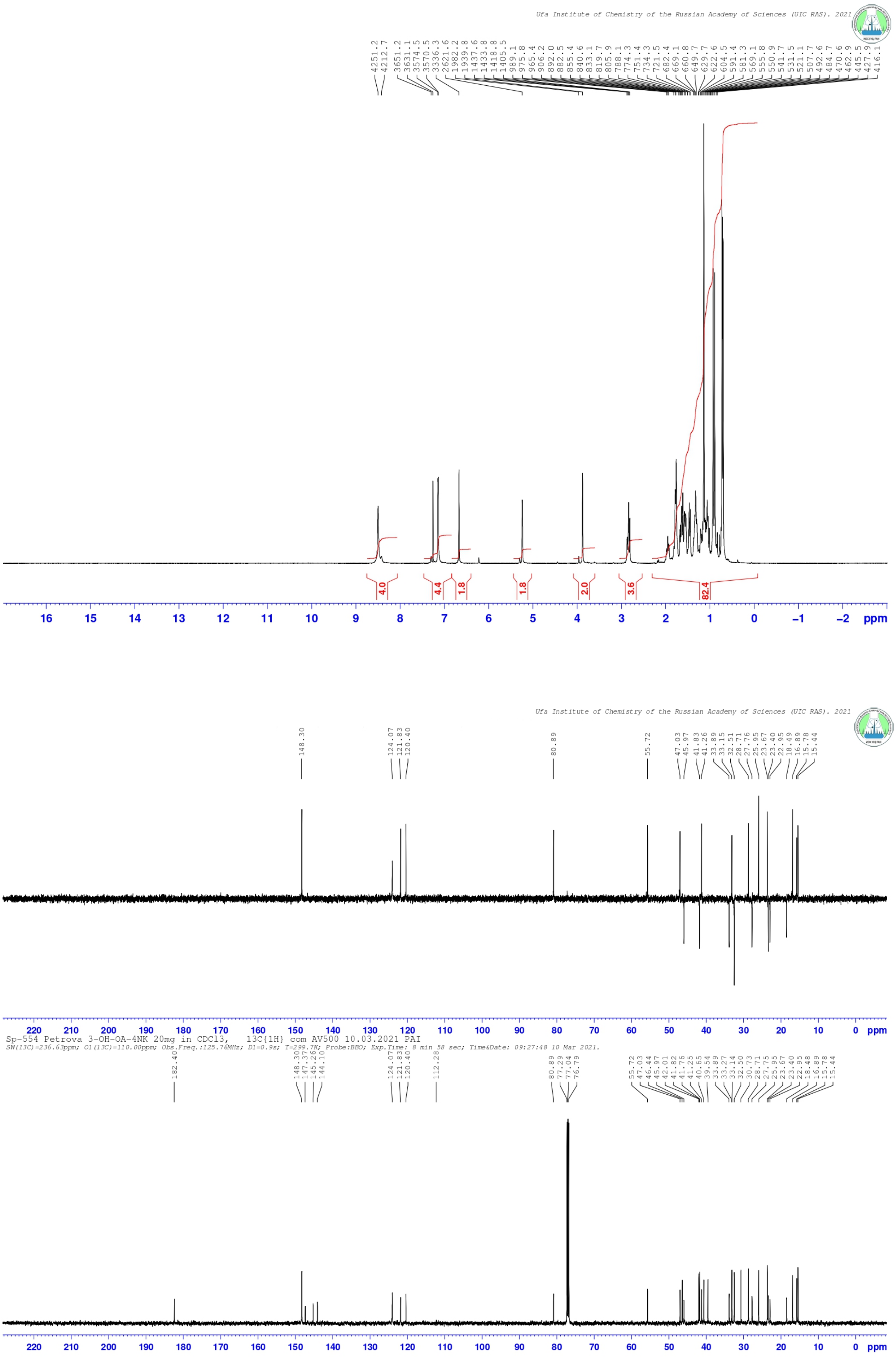

Figure S24. MS-APSI spectra of compound **16**

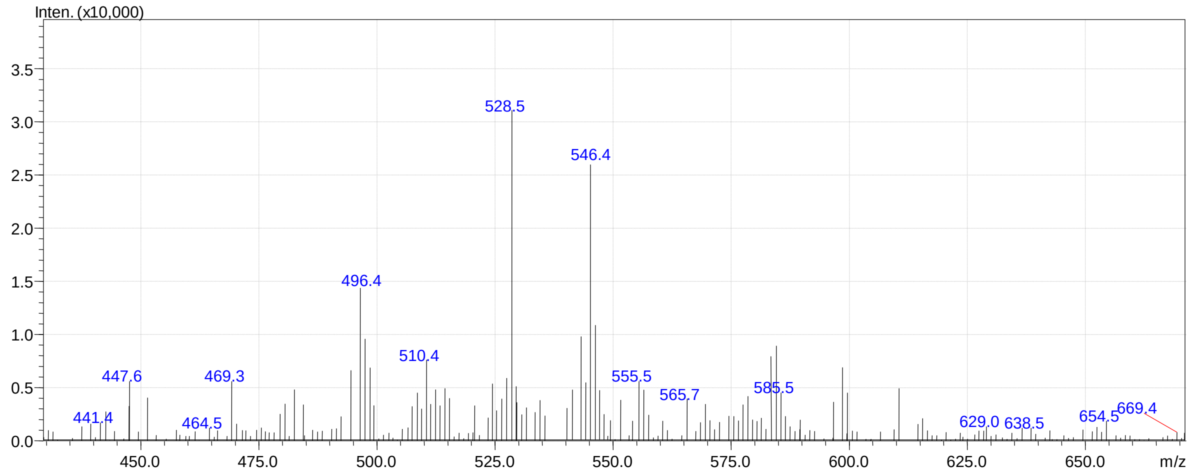

Figure S25. ^{1}H and $^{13}C\{^{1}H\}$ (DEPT-135 edited) NMR spectra of compound **17**

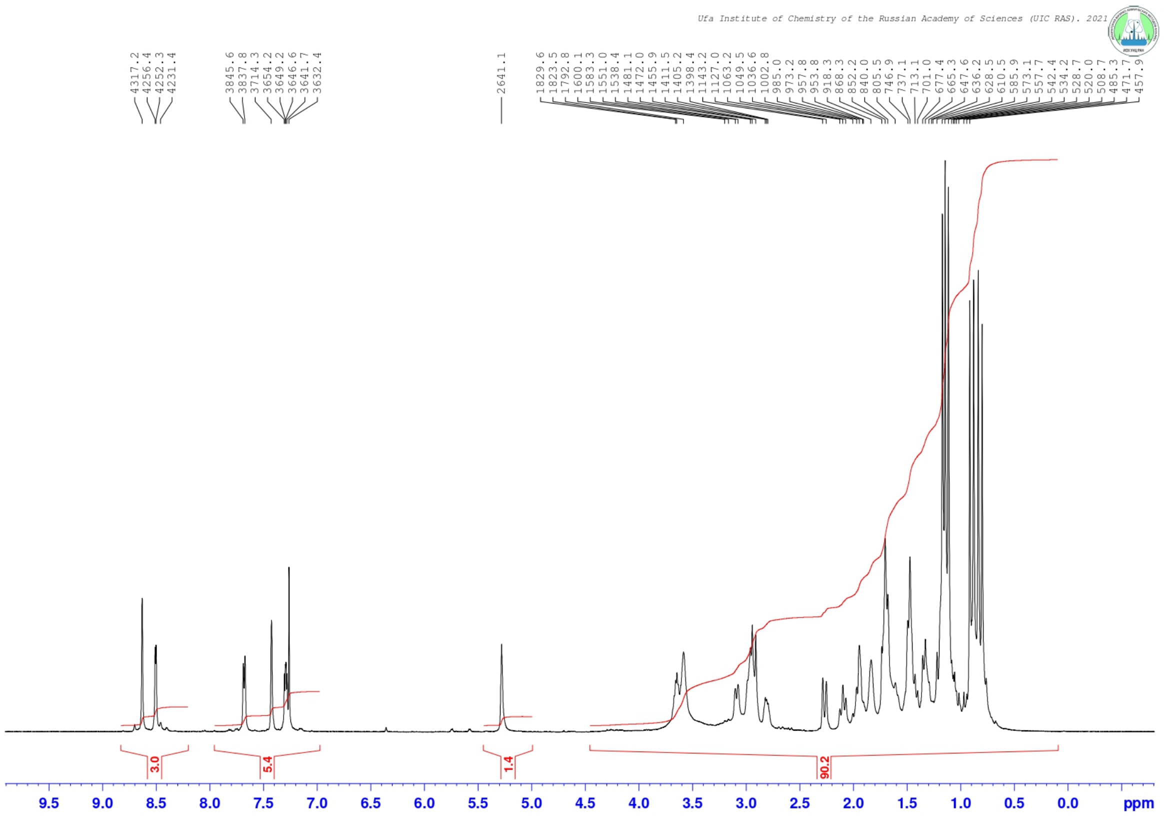

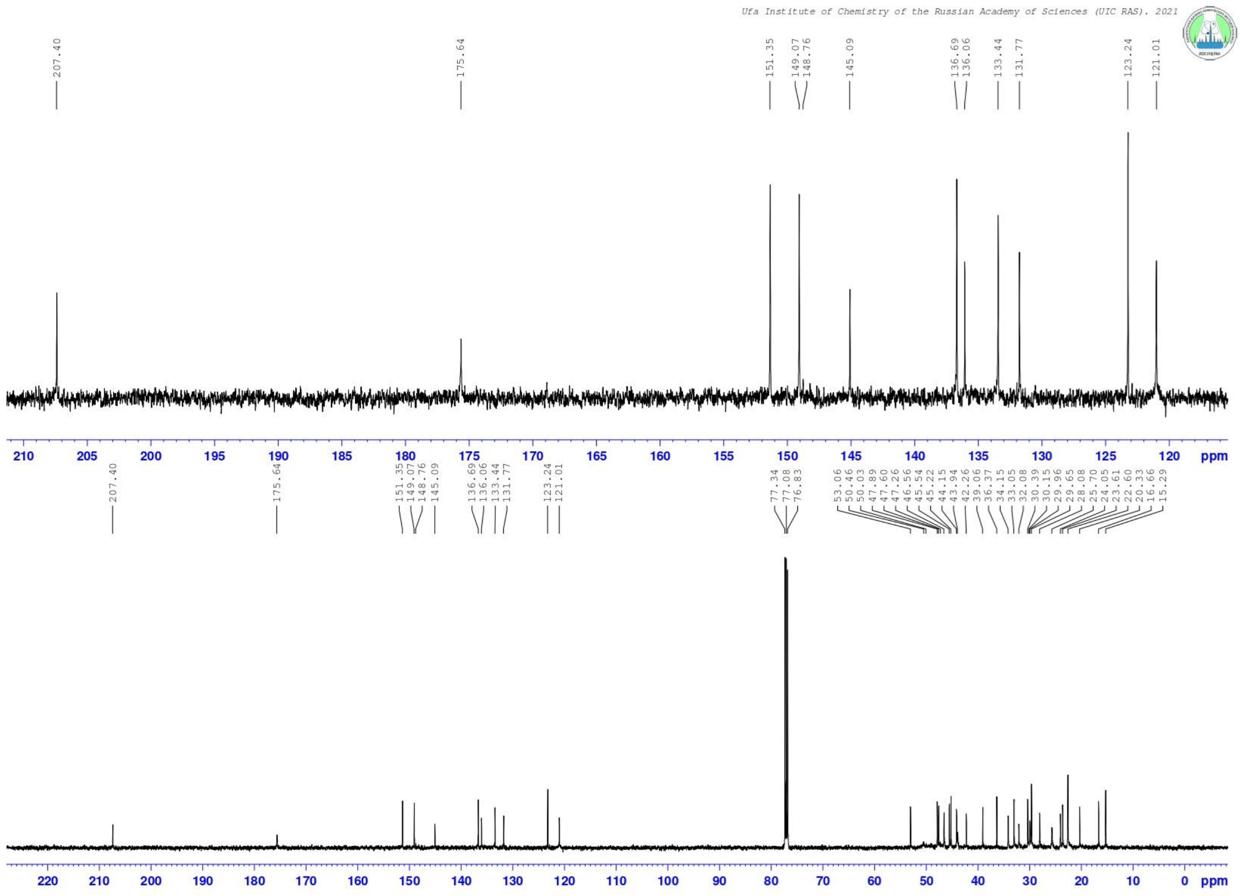

Figure S26. MS-APSI spectra of compound **17**

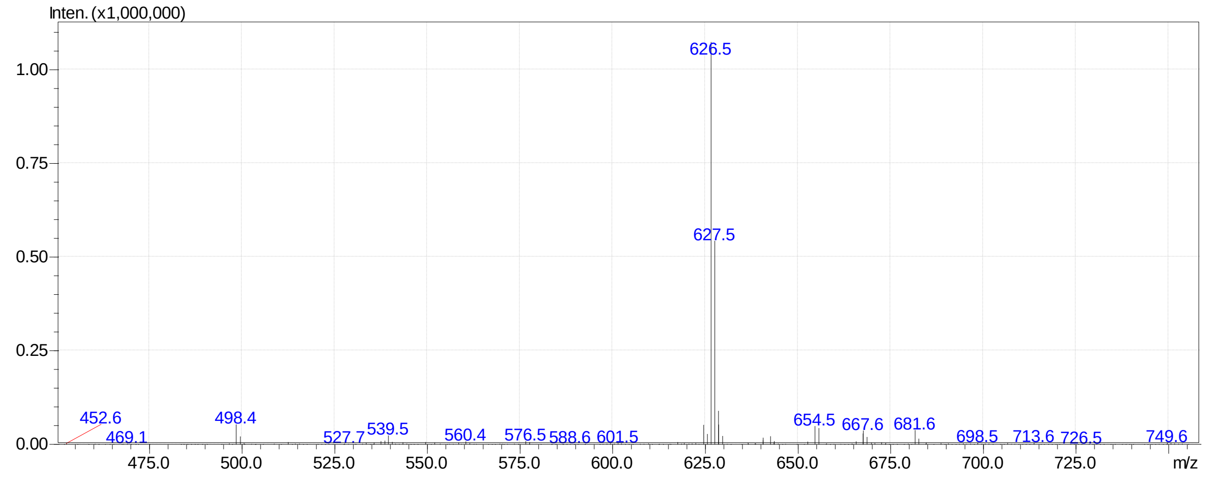

Figure S27. ^{1}H and $^{13}C\{^{1}H\}$ NMR spectra of compound **18**

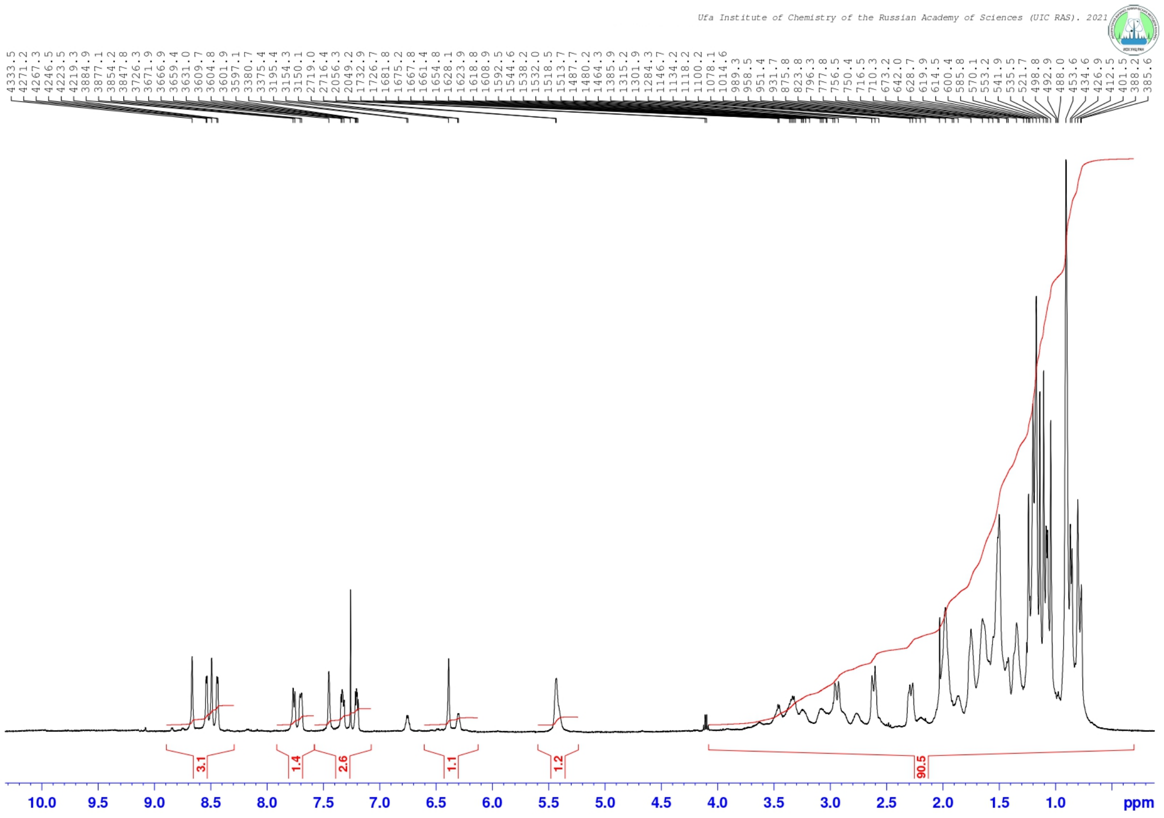

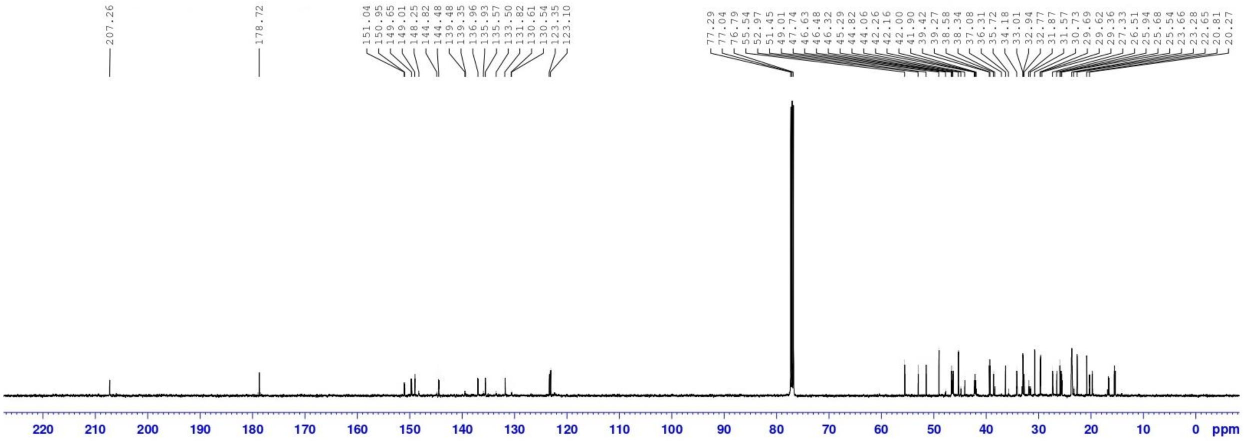

Figure S28. MS-APSI spectra of compound **18**

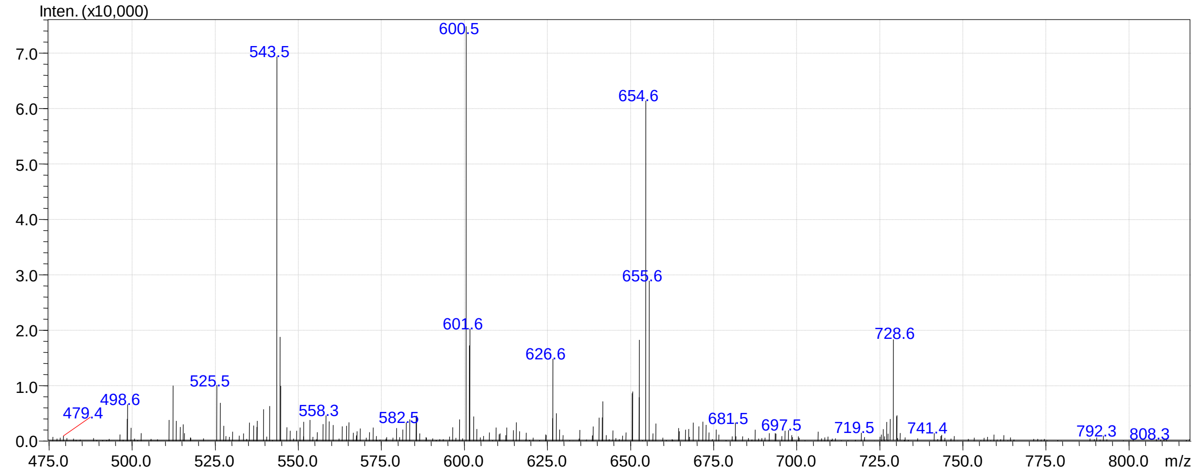

Figure S29. ^{1}H and $^{13}C\{^{1}H\}$ NMR spectra of compound **19**

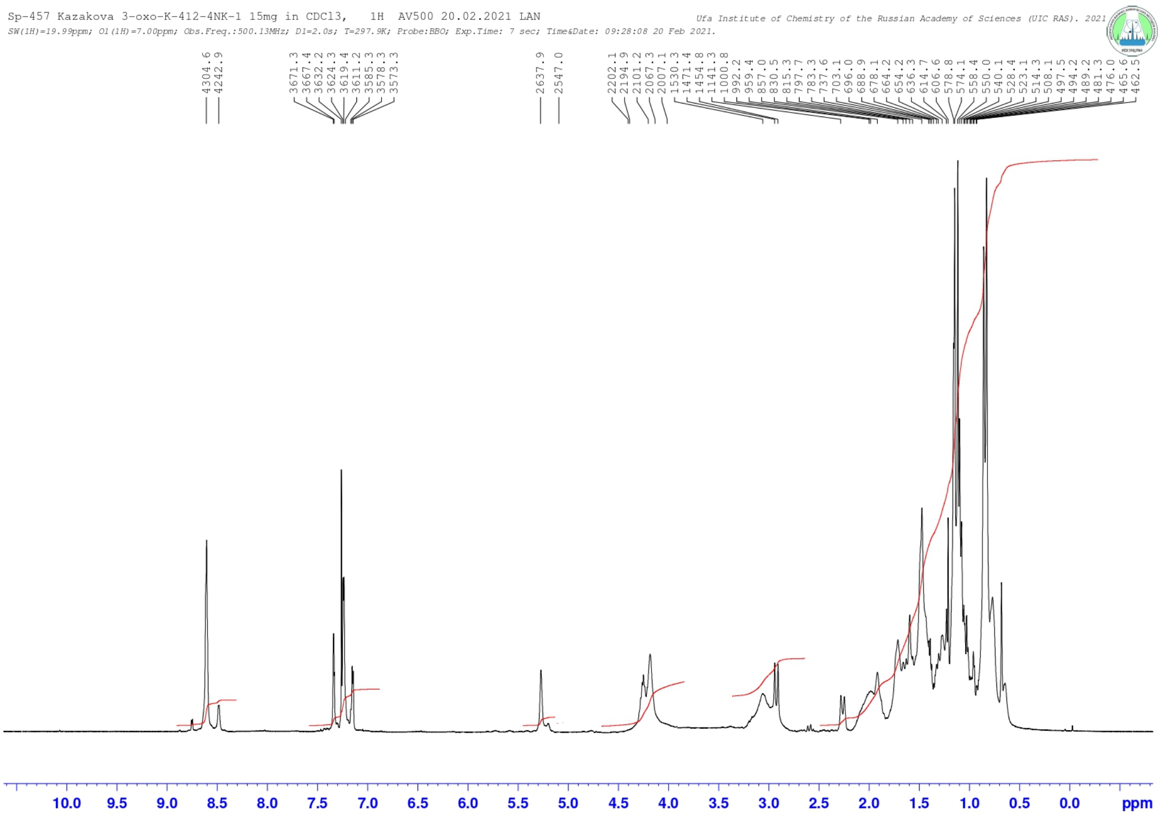

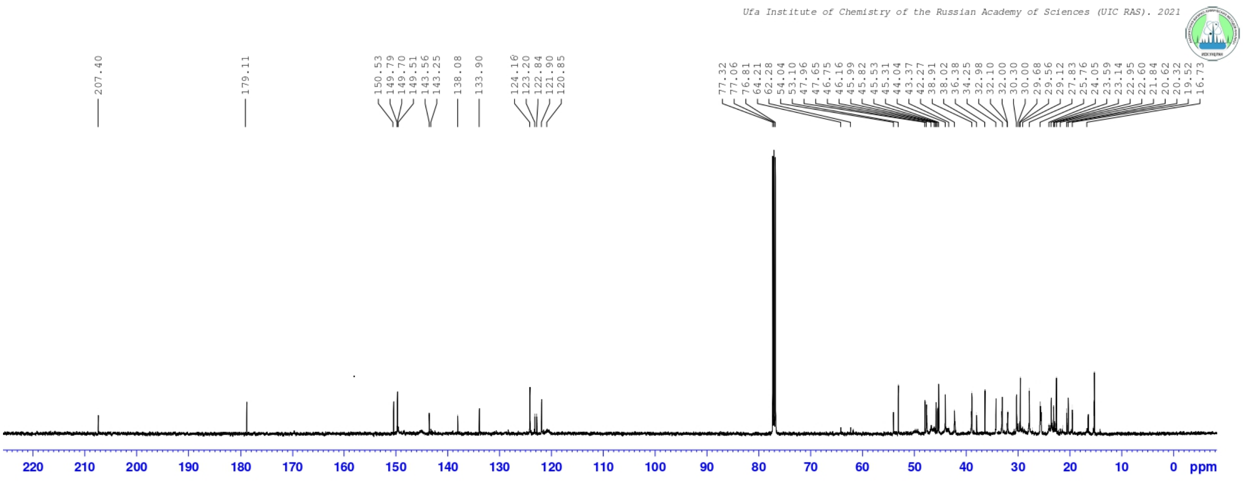

Figure S30. MS-APSI spectra of compound **19**

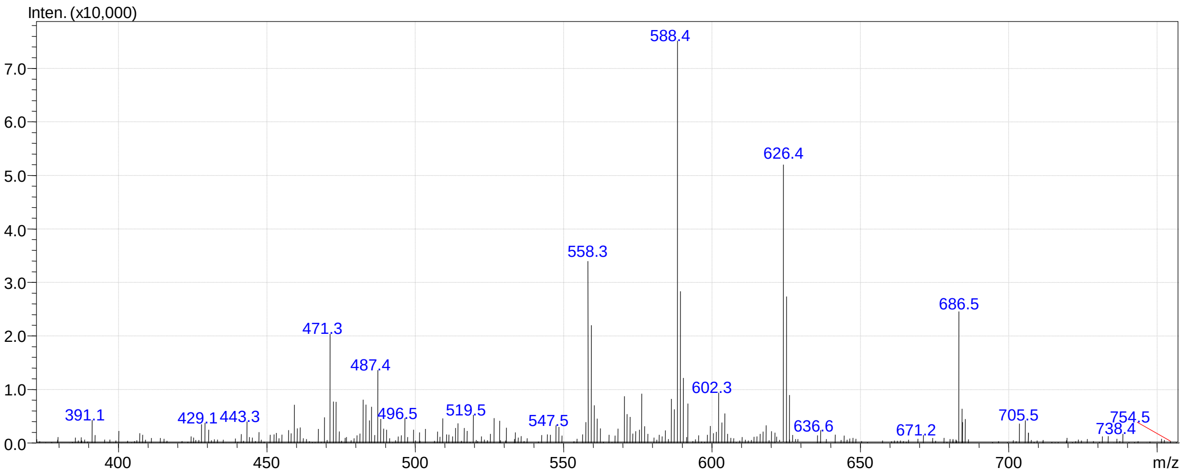
Inten. (x10,000)
7.0
6.0
5.0
4.0
3.0
2.0
1.0
0.0
400
450
500
550
600
650
700
m/z
588.4
626.4
558.3
686.5
471.3
487.4
602.3
391.1
429.1
443.3
496.5
519.5
547.5
636.6
671.2
705.5
754.5
738.4

Figure S31. ^{1}H and $^{13}C\{^{1}H\}$ NMR spectra of compound **20**

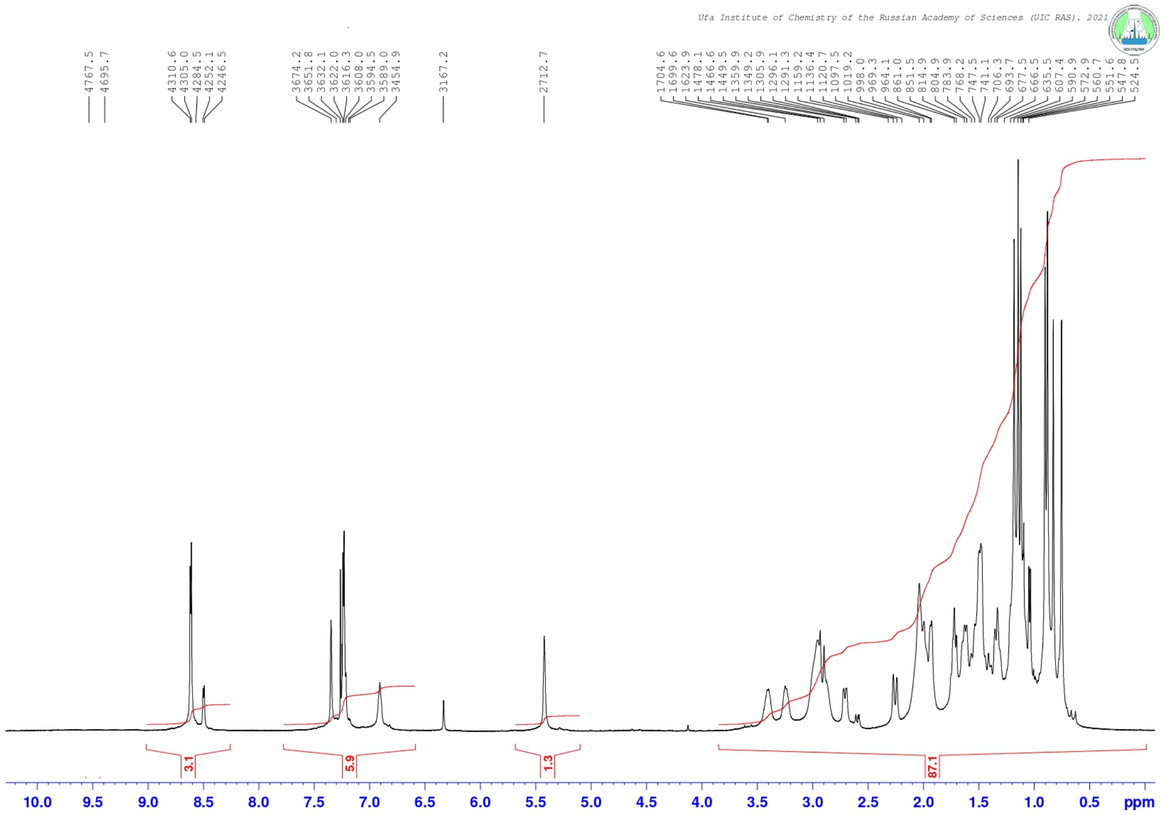

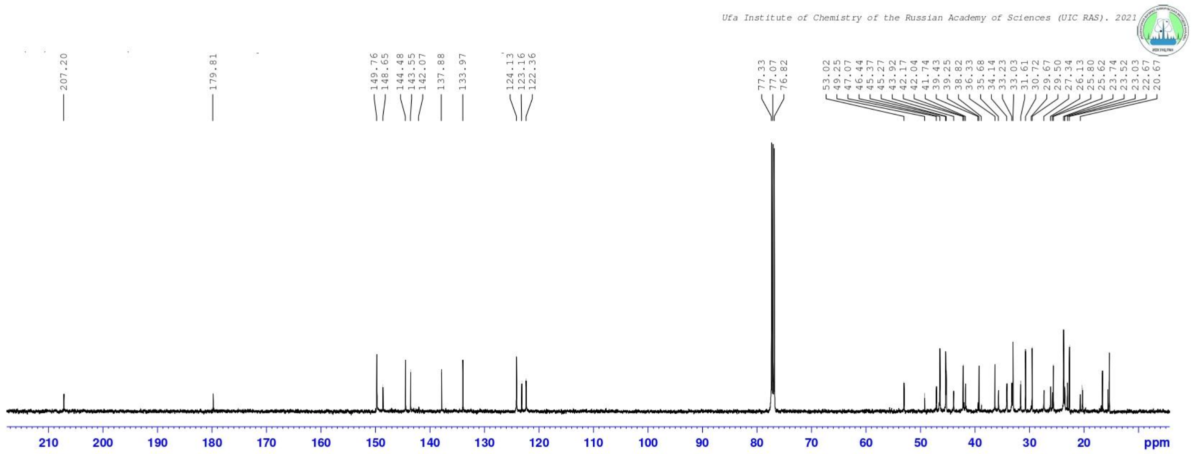

Figure S32. MS-APSI spectra of compound **20**

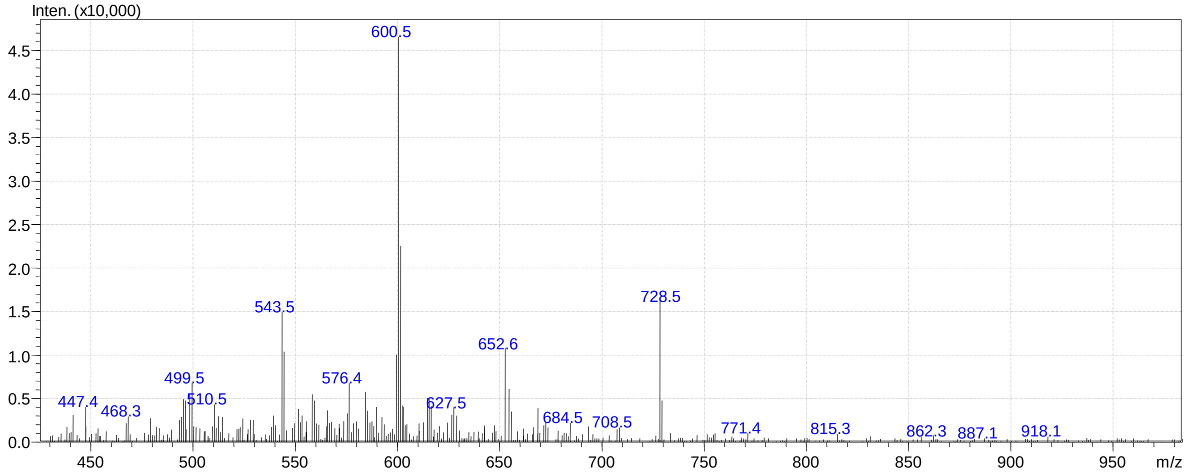

Figure S33. ^{1}H and ^{13}C{^{1}H} NMR spectra of compound **22**

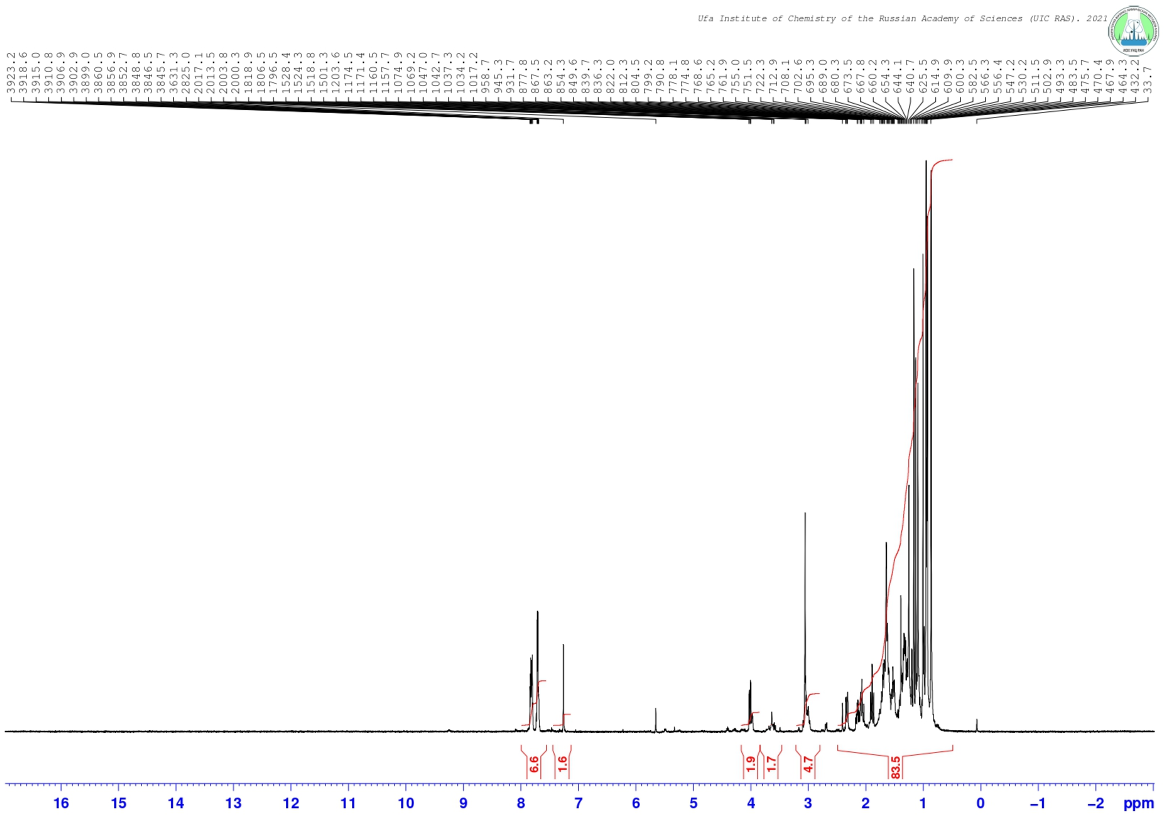

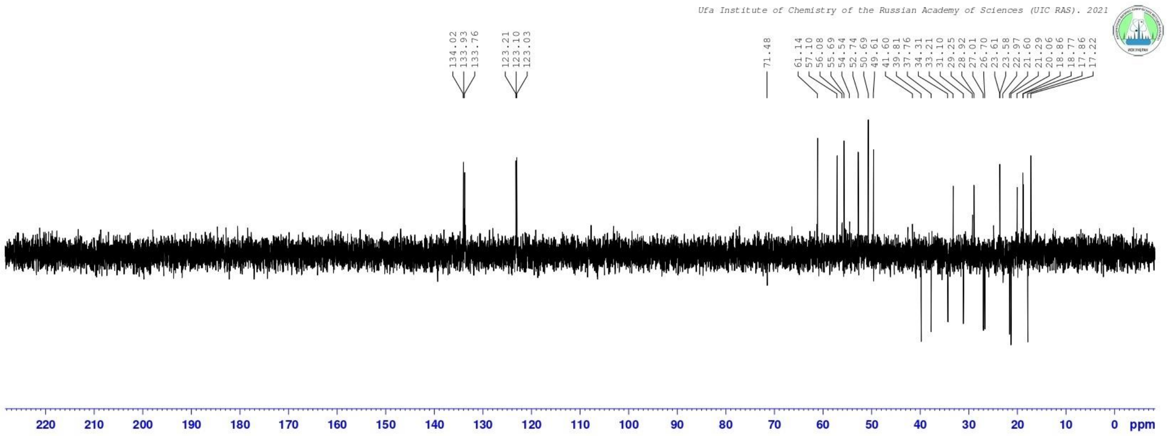

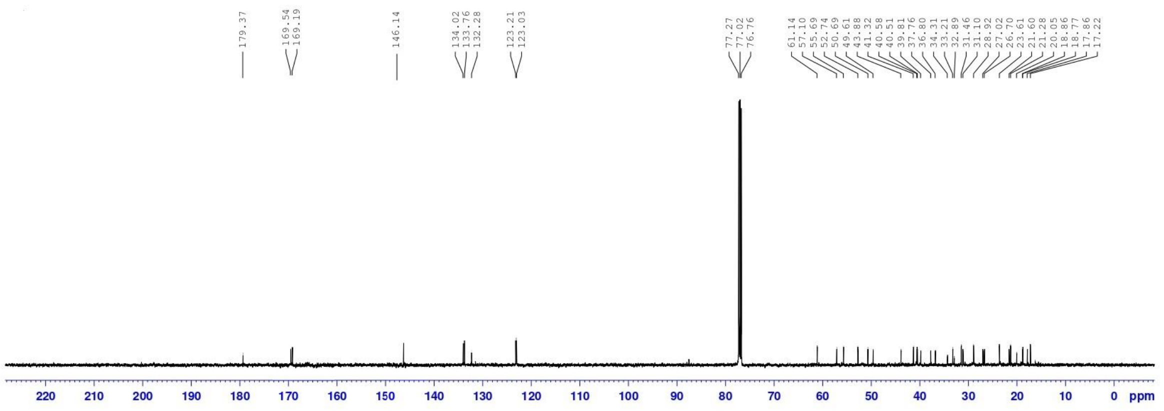

Figure S34. MS-APSI spectra of compound **22**

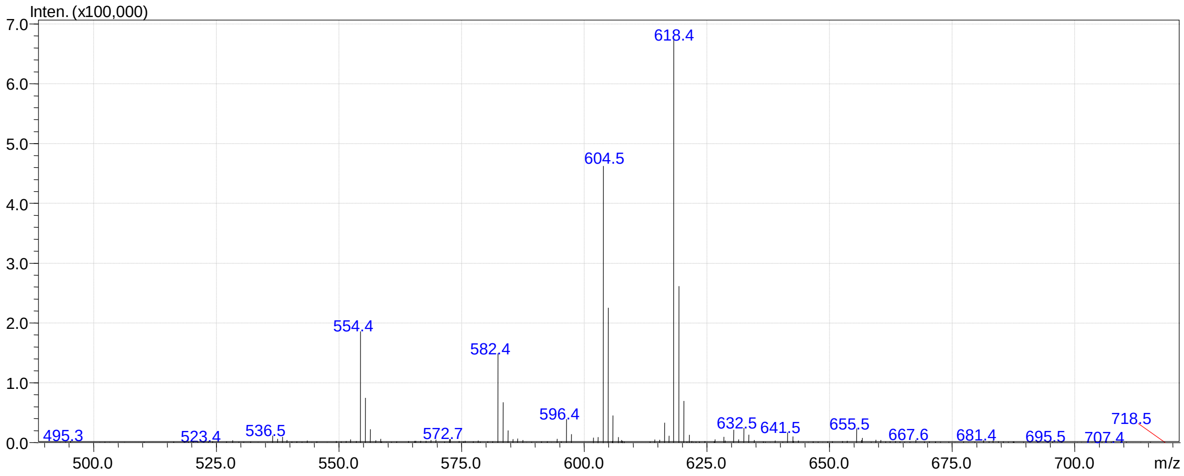

Supplement: Supplementary file 1 [file molecules-27-08499-s001.zip › molecules-2023904-supplementary.pdf]
